# Hypoxia-inducible factor-1α plays roles in Epstein-Barr virus’s natural life cycle and tumorigenesis by inducing lytic infection through direct binding to the immediate-early *BZLF1* gene promoter

**DOI:** 10.1371/journal.ppat.1006404

**Published:** 2017-06-15

**Authors:** Richard J. Kraus, Xianming Yu, Blue-leaf A. Cordes, Saraniya Sathiamoorthi, Tawin Iempridee, Dhananjay M. Nawandar, Shidong Ma, James C. Romero-Masters, Kyle G. McChesney, Zhen Lin, Kathleen R. Makielski, Denis L. Lee, Paul F. Lambert, Eric C. Johannsen, Shannon C. Kenney, Janet E. Mertz

**Affiliations:** 1 McArdle Laboratory for Cancer Research, Department of Oncology, University of Wisconsin-Madison School of Medicine and Public Health, Madison, Wisconsin, United States of America; 2 National Nanotechnology Center, National Science and Technology Development Agency, Thailand Science Park, Pathum Thani, Thailand; 3 Department of Pathology, Tulane University Health Sciences Center and Tulane Cancer Center, New Orleans, Louisiana, United States of America; 4 Department of Medicine, University of Wisconsin-Madison School of Medicine and Public Health, Madison, Wisconsin, United States of America; Baylor College of Medicine, UNITED STATES

## Abstract

When confronted with poor oxygenation, cells adapt by activating survival signaling pathways, including the oxygen-sensitive transcriptional regulators called hypoxia-inducible factor alphas (HIF-αs). We report here that HIF-1α also regulates the life cycle of Epstein-Barr virus (EBV). Incubation of EBV-positive gastric carcinoma AGS-Akata and SNU-719 and Burkitt lymphoma Sal and KemIII cell lines with a prolyl hydroxylase inhibitor, L-mimosine or deferoxamine, or the NEDDylation inhibitor MLN4924 promoted rapid and sustained accumulation of both HIF-1α and lytic EBV antigens. ShRNA knockdown of HIF-1α significantly reduced deferoxamine-mediated lytic reactivation. HIF-1α directly bound the promoter of the EBV primary latent-lytic switch *BZLF1* gene, Zp, activating transcription via a consensus hypoxia-response element (HRE) located at nt -83 through -76 relative to the transcription initiation site. HIF-1α did not activate transcription from the other EBV immediate-early gene, *BRLF1*. Importantly, expression of HIF-1α induced EBV lytic-gene expression in cells harboring wild-type EBV, but not in cells infected with variants containing base-pair substitution mutations within this HRE. Human oral keratinocyte (NOK) and gingival epithelial (hGET) cells induced to differentiate by incubation with either methyl cellulose or growth in organotypic culture accumulated both HIF-1α and Blimp-1α, another cellular factor implicated in lytic reactivation. HIF-1α activity also accumulated along with Blimp-1α during B-cell differentiation into plasma cells. Furthermore, most *BZLF1*-expressing cells observed in lymphomas induced by EBV in NSG mice with a humanized immune system were located distal to blood vessels in hypoxic regions of the tumors. Thus, we conclude that HIF-1α plays central roles in both EBV’s natural life cycle and EBV-associated tumorigenesis. We propose that drugs that induce HIF-1α protein accumulation are good candidates for development of a lytic-induction therapy for treating some EBV-associated malignancies.

## Author summary

Most adults throughout the world are infected with Epstein-Barr virus (EBV), a human herpesvirus frequently associated in a latent state with some cancers of epithelial and B-cell origin such as nasopharyngeal carcinoma and Burkitt lymphoma, respectively. To develop an oncolytic therapy for treating patients with EBV-associated cancers, we need a method to efficiently induce synthesis of lytic EBV proteins. The EBV protein encoded by its immediate-early *BZLF1* gene usually mediates the switch into lytic viral infection. We show here that HIF-1α, a cellular transcription factor that accumulates in cells when deprived of normal levels of oxygen, can induce lytic EBV infection. HIF-1α mediates this switch by directly binding to a specific sequence located within the *BZLF1* gene promoter, activating its expression. Importantly, we also show that deferoxamine, an FDA-approved drug that inhibits degradation of HIF-1α, can induce synthesis of lytic EBV proteins in some EBV-positive epithelial and lymphocytic cell lines. These findings indicate that HIF-1α-stabilizing drugs, administered in combination with nucleoside analogues such as ganciclovir, may be helpful as part of a lytic-induction therapy for treating some patients with EBV-positive malignancies.

## Introduction

Epstein-Barr virus (EBV) is a ubiquitous human gamma herpesvirus that infects over 90% of the world’s population. In healthy hosts, primary infection after childhood often results in infectious mononucleosis (IM). Following primary infection, EBV establishes a life-long latent infection in a tiny subset of its host’s memory B cells where its genome is maintained as an episome that replicates in synchrony with the host’s cellular DNA (reviewed in [1,2]). Latency is characterized by expression of, at most, a small number of viral protein-encoding genes (EBNAs and LMPs), two non-coding RNAs (EBERs), and some micro (mi) RNAs (reviewed in [3]). Latent EBV infection is associated with some malignancies in humans, including nasopharyngeal carcinoma (NPC), some gastric cancers (GC), a subset of Burkitt lymphomas (BL), diffuse large B-cell lymphomas (DLBCL), and post-transplant lymphoproliferative diseases (PTLD) (reviewed in [1,4,5]). Several EBV-encoded latency proteins and miRNAs have been shown to contribute to cell transformation and tumorigenesis [1,3].

Like other herpesviruses, EBV’s long-term success requires it to undergo lytic as well as latent modes of infection during its life cycle. While latent infection permits persistence of the virus for the life of the host, lytic replication enables production of infectious virus necessary for transmission from cell to cell and host to host. Thus, EBV occasionally reactivates out of latently infected B cells. Physiological inducers of EBV reactivation include B-cell antigen receptor (BCR) activation leading to plasma cell differentiation [2], butyrate [6,7], and transforming growth factor β (TGF-β) [8,9]. Subsequently, EBV infects differentiated cells within the normal oropharyngeal epithelial where infection is usually lytic [1,2,10].

EBV reactivation is initiated by transcriptional activation of one or both of the viral immediate-early (IE) gene promoters, Zp and Rp, leading to production of its two IE proteins, Zta (the product of the *BZLF1* gene; also called Z, ZEBRA, and EB1) and Rta (the product of the *BRLF1* gene; also called R), respectively. Synthesis of Zta is sufficient to induce reactivation in most EBV-positive (EBV^+^) cell lines [11], while Rta induces reactivation in some cell lines [12,13]. Rta and Zta are transcription factors that then activate each other’s promoters [12,14,15] and, subsequently, activate expression of EBV’s early (E) genes, including *BMRF1*, a viral DNA polymerase processivity factor [also called early-antigen diffuse (EAD)], and *BGLF4*, a virus-encoded protein kinase (reviewed in [16]).

Given that expression of the *BZLF1* gene serves as the primary gatekeeper to the viral latent-to-lytic switch in most EBV^+^ cell lines, transcriptional regulation of Zp has been studied extensively. Numerous *cis*-acting elements and their cognate *trans*-acting factors have been identified that contribute to silencing during latency and activation in response to inducers (reviewed in [16]).

Poor oxygenation, *i*.*e*., hypoxia, contributes to tumor progression and resistance to conventional chemotherapy (reviewed in [17–19]). The mechanisms by which cells respond to hypoxic environments are known (reviewed in [20,21]). Under normal oxygen tension corresponding to approximately 21% O_2_, cellular transcription factors called hypoxia-inducible factor alphas (HIF-αs) are synthesized but rapidly degraded via the ubiquitin-dependent proteasome pathway. Three distinct genes encode the HIF-αs (HIF-1α, HIF-2α, and HIF-3α). Hydroxylation of specific proline residues by oxygen-dependent cellular prolyl hydroxylases (*e*.*g*., PHD2, encoded by the *EGLN1* gene) marks these proteins for ubiquitin-mediated degradation. The hydroxylation reaction catalyzed by PHDs also involves the conversion of α–ketoglutarate to succinate, Fe^2+^ to Fe ^3+^, and O_2_ to CO_2_, with vitamin C required for the regeneration of Fe^2+^. Under hypoxic conditions (or in the presence of iron chelators or competitors), PHDs fail to hydroxylate HIF-αs, resulting in accumulation of these proteins to high levels. Stabilized HIF-αs form heterodimers with their constitutively present binding partner, HIF-1β [also called aryl hydrocarbon nuclear receptor translocator (ARNT)], translocate to the nucleus, and sequence-specifically bind to hypoxia-response elements (HREs) located within the promoter regions of cellular genes involved in angiogenesis, anaerobic metabolism, and erythropoiesis.

The roles hypoxia and HIF-1α play in the life cycle of Kaposi’s sarcoma herpesvirus (KSHV), another member of the gamma herpesvirus family, have been extensively studied (reviewed in [22,23]). Analogously, Jiang *et al*. [24] reported that incubation of the EBV^+^ marmoset-derived B-cell line, B95-8, in 2% oxygen conditions leads to induction of Zta synthesis within one-to-two days, and Murata *et al*. [25] confirmed that hypoxia (1% oxygen; 36 h) induces *BZLF1* gene expression in human EBV^+^ Akata B cells and LCLs as well as B95-8 cells.

Here, we report that drugs that mimic hypoxia induce lytic EBV infection in some EBV^+^ epithelial and B-cell lines by a HIF-1α-dependent mechanism. HIF-1α induces the switch to lytic-gene expression through directly activating *BZLF1* gene expression by sequence-specific binding to an HRE located within Zp. We further show that HIF-1α can play important roles in EBV’s natural life cycle and tumorigenesis induced by this virus. These findings suggest a new class of drugs that may be useful in the development of a lytic-induction therapy for treating patients with some EBV-associated malignancies.

## Results

### HIF-α stabilizers induce EBV reactivation in some EBV^+^ cell lines

**Fig 1:**
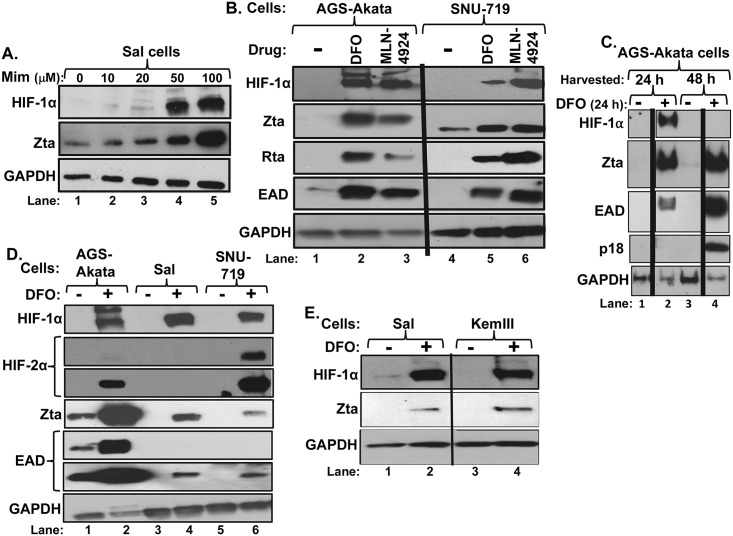
Hypoxia mimics induce accumulation of lytic EBV proteins in some EBV^+^ cell lines. (A)) Immunoblots showing accumulation of HIF-1α and Zta after incubation of Sal cells with the indicated concentrations of L-Mimosine for 24 h. (B) Immunoblots showing relative levels of HIF-1α and the indicated lytic EBV antigens present in AGS-Akata and SNU-719 cells 24 h after addition to the medium of DFO (200 μM; lanes 2 and 5), MLN4924 (5 μM; lanes 3 and 6), or the diluent vehicle (lanes 1 and 4). (C) Immunoblots showing accumulation of HIF-1α and the EBV-encoded proteins Zta, EAD, and VCA/p18 after incubation of AGS-Akata cells with (+) or without (-) 200 μM DFO for 24 h followed by immediate harvesting (lanes 1 and 2) or incubation for an additional 24 h in the absence of DFO prior to harvesting (lanes 3 and 4). (D) Immunoblots showing the relative levels of accumulation of the indicated proteins in several cells lines incubated in the presence (+) or absence (-) of 200 μM DFO for 24 h. (E) Immunoblots comparing accumulation of HIF-1α and Zta in Sal versus KemIII cells after incubation as described in panel D. Whole-cell extracts were prepared, processed, and probed for the indicated proteins. GAPDH served as a loading control. Data shown are representative of numerous independent sets of experiments.

Our long-term objective is to find drugs suitable for use in EBV-targeted oncolytic therapy [26,27]. Thus, we chose to mimic hypoxia by incubating cells with deferoxamine (DFO; also called Desferal) or L-mimosine (Mim; also called Leucenol), two drugs that inhibit prolyl hydroxylase activity by chelating iron [28]. The EBV^+^ cell lines examined were Burkitt lymphoma-derived Sal and KemIII and gastric carcinoma-derived AGS-Akata and SNU-719. SNU-719, Sal, and KemIII retain their original-infecting EBV genomes. SNU-719 cells have type I latency plus LPM2A, Sal cells have Wp-restricted latency, and KemIII have type III latency. In an initial experiment, we found that incubation of Sal cells with mimosine promoted both stabilization of HIF-1α and induction of synthesis of the immediate-early (IE) lytic EBV antigen, Zta (Fig 1A). However, because mimosine is not FDA-approved for internal use, we largely focused on DFO in subsequent experiments. Incubation of all four of these cell lines with DFO for 24 h promoted stabilization of HIF-1α protein along with inducing synthesis of Zta (Fig 1B–1E). Quantitation of the efficiency of EBV reactivation by staining cells for presence of Zta indicated that 15%-30% of AGS-Akata cells were induced into lytic-gene expression within 24 h of addition of 200 μM DFO, while 1 ½%-to-3% of Sal, SNU-719, and KemIII cells were induced within this time frame (S1 Fig; Fig 1D and 1E).

MLN4924 (Pevonedistat), an inhibitor of the NEDD8-activating enzyme (NAE), also blocks degradation of HIF-αs. By preventing NEDDylation of the cullin-RING E3 ubiquitin ligases (CRLs), MLN4924 inhibits degradation of approximately 20% of cellular proteins, including the HIF-αs, whose levels are regulated in part via the proteasome degradation pathway (reviewed in [29]). We found that MLN4924 reactivation of EBV into lytic infection at a roughly similar efficiency to DFO in the EBV^+^ GC-derived cell lines (Fig 1B). Thus, we conclude that two classes of HIF-α stabilizing drugs with different off-target effects can both induce lytic EBV infection.

We also asked whether temporary stabilization of HIF-αs resulted in abortive lytic infection or activation of the complete lytic replication cycle. Removal of DFO after 24 h led to loss of HIF-1α, as expected, yet the lytic cycle continued to progress, leading to high-level synthesis of EAD and the late (L) EBV-encoded viral capsid antigen (VCA, also called p18) by 48 h (Fig 1C). These data also suggest that the percentage of cells reactivated by DFO might well be higher than observed after only 24 h. Thus, we conclude that HIF-α-stabilizing drugs can induce lytic EBV infection in EBV^+^ cells of lymphocytic and epithelial origin and in a variety of latency types.

### HIF-1α is the predominant HIF-α expressed in EBV^+^ cells

While DFO induced high-level accumulation of HIF-1α protein in all four of these cell lines, it only induced HIF-2α protein to moderately high levels in SNU-719 cells (Fig 1D). Analysis of RNA-sequencing data of mRNA purified from SNU-719 cells indicated that HIF-1α mRNA was 5.5-fold more abundant than HIF-2α mRNA and 43-fold more abundant than HIF-3α mRNA. Thus, even though we could detect some HIF-2α protein in DFO-treated SNU-719 and AGS-Akata cells, it was probably present at considerably lower levels than was HIF-1α protein.

HIF-1α was also the predominant isoform of the three HIF-αs detected at the RNA level in primary tissues exhibiting EBV tropism. Transcriptome analysis of four high EBV^+^ primary gastric cancers from the TCGA cohort [30] indicated that HIF-1α mRNA was, on average, 2.9-fold more abundant than HIF-2α mRNA (range 1.9-fold to 4.9-fold), with HIF-3α mRNA undetectable above background level. A similar analysis of 17 EBV^+^ endemic Burkitt lymphomas [31] indicated HIF-1α mRNA was, on average, nine-fold more abundant than HIF-2α mRNA (range 2-fold to 19-fold), with HIF-3α mRNA detectable above background in only one of these 17 tumors (at 1/20^th^ of the HIF-1α level). Further, HIF-1α accounts for almost all of the HIF-α-related mRNA present in primary human B-cells throughout the various stages of B-cell differentiation into plasma cells (*e*.*g*., see data presented below). Thus, although HIF-2α may contribute to EBV’s life cycle under some conditions in epithelial cells, HIF-1α appears to be the predominant HIF-α expressed in cell types of physiological relevance to EBV. Given this finding, most of the studies presented here were performed with HIF-1α. We occasionally confirmed our findings with HIF-2α and did not conduct further studies with HIF-3α.

### DFO efficiently induces EBV reactivation in a subset of HIF-1α^+^ cells

**Fig 2:**
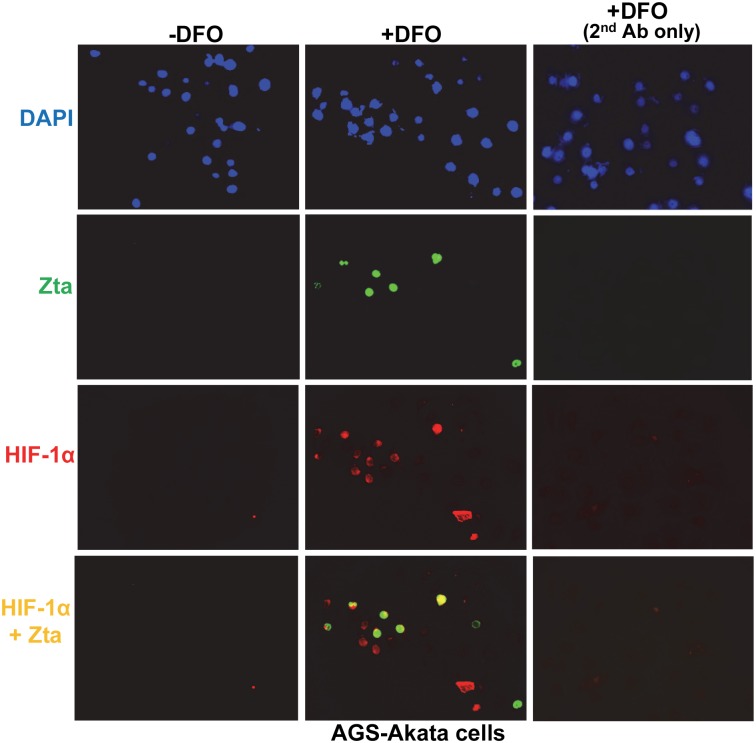
Dual immunofluorescence staining indicates DFO efficiently induces synthesis of Zta protein in a subset of HIF-1α-expressing EBV-positive cells. AGS-Akata cells grown on cover slips were incubated for 24 h in the absence (-) or presence (+) of 200 μM DFO prior to fixing and processing for co-detection by IFS of the proteins Zta (green) and HIF-1α (red). DFO-treated cells were independently probed with the green-conjugated secondary antibody absent primary antibodies to control for background GFP encoded by the virus.

If HIF-1α induces EBV reactivation, one would expect most Zta^+^ cells to also be HIF-1α^+^. To determine the level of coincidence between Zta^+^ and HIF-1α^+^ cells, we performed dual immunofluorescence staining (IFS) assays. Consistent with our hypothesis, we found that almost all of the Zta^+^ cells were also HIF-1α^+^ in AGS-Akata cells that had been incubated with DFO for 24 h (Fig 2). The occasional Zta^+^, HIF-1α-negative cell we observed was likely the consequence of AGS-Akata cells exhibiting some spontaneous reactivation (*e*.*g*., S1B Fig). Thus, we conclude that DFO efficiently induces EBV reactivation in AGS-Akata cells, at least in part, by stabilizing HIF-1α.

### DFO induction of Zta synthesis is mediated primarily via HIF-1α

**Fig 3:**
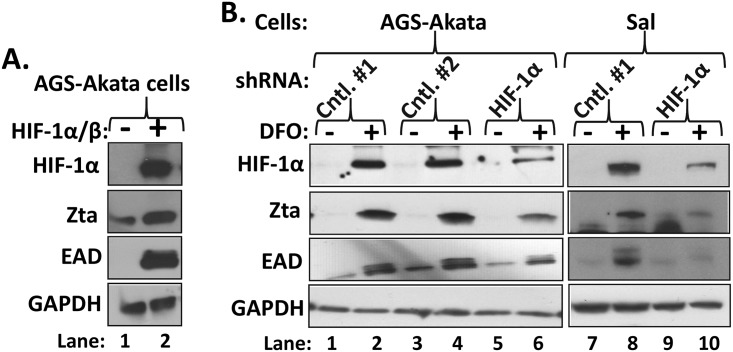
HIF-1α addition is sufficient to induce EBV reactivation and necessary for efficient induction by DFO. (A) Immunoblots showing addition of HIF-1α is sufficient to induce lytic EBV reactivation. AGS-Akata cells grown in 10-cm dishes were transfected with 1 μg each of pHA-HIF-1α P402A/P564A-pcDNA3 plus pHIF-β (+) or 2 μg of their empty vector, pcDNA3, as a control (-) and incubated for 48 h prior to preparation of whole-cell extracts. Data are representative of numerous independent experiments. (B) Immunoblots showing knockdown of HIF-1α inhibits DFO-induced synthesis of EBV lytic antigens. Lanes 1–6, AGS-Akata cells maintained in 10-cm dishes were co-transfected with 0.8 μg of each of five lentiviruses that express different shRNAs targeting HIF-1α (lanes 5–6) or 4 μg of a lentivirus that expresses the non-targeting shRNA cntl. #1 or cntl. #2 (lanes 1–2 and lanes 3–4, respectively). Two days later, the cells were incubated in the absence (-) or presence (+) of 200 μM DFO for 24 h prior to harvesting and preparation of whole-cell extracts. Lanes 7–10, Sal cells were infected with the indicated packaged lentiviruses; three days later, the cells were incubated in the absence (-) or presence (+) of 200 μM DFO for 24 h and processed likewise. Data are representative of two independent experiments. GADPH served as a loading control.

To demonstrate a direct causal role of HIF-1α in reactivation, we evaluated induction of synthesis of lytic EBV antigens after addition of HIF-1α. AGS-Akata cells were co-transfected with: (i) a plasmid expressing an oxygen-insensitive variant of HIF-1α that contains alanine substitutions in the proline residues targeted for hydroxylation by PHDs; and (ii) a plasmid expressing HIF-1α’s heterodimeric partner, HIF-1β/ARNT. Addition of HIF-1α/HIF-1β was sufficient to strongly induce synthesis of Zta and EAD (Fig 3A).

We also performed a reciprocal experiment. Knockdown of HIF-1α expression by 80%-90% in AGS-Akata cells resulted in a comparable level of loss of DFO-induced synthesis of Zta and EAD (Fig 3B, lanes 1–6). Similar findings were observed in Sal cells infected with these lentiviruses (Fig 3B, lanes 7–10). Thus, we conclude that DFO-directed induction of lytic EBV infection is mediated largely by HIF-1α.

### HIF-1α primarily induces EBV lytic-gene expression by activating transcription from Zp

**Fig 4:**
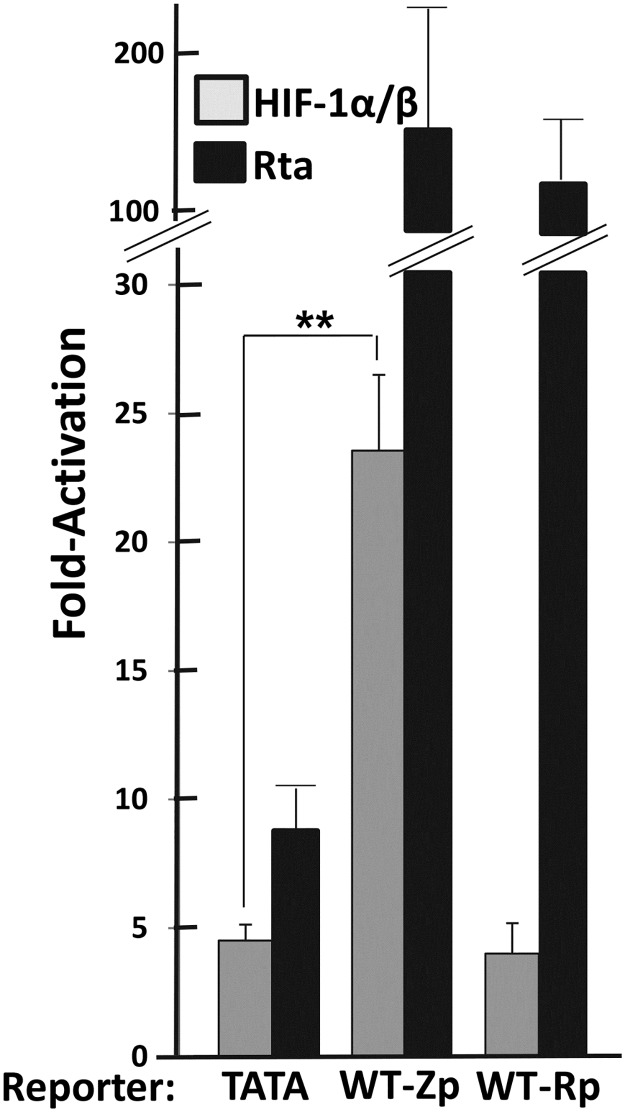
HIF-1α induces transcriptional activation from Zp, but not Rp. 293T cells maintained in 24-well plates were co-transfected with (i) 200 ng DNA of a pGL3-Basic luciferase reporter containing the nt -30 to +30 region of the HSV TK gene (pTATA-luc) as a control, the nt -221 to +30 region of Zp (pWTZp-luc), or the nt -1069 to +38 region of Rp (pWTRp-luc), and (ii) pHA-HIF-1αP402A/P564A-pcDNA3 plus pHIF-1β (40 ng each), pcDNA3-BRLF1 (30 ng) as a positive control, or pcDNA3 (80 ng) as a negative control. Cells were harvested 48 h later, and luciferase activities were determined. Data obtained with each reporter were normalized to the value obtained when co-transfected with pcDNA3; they are averages from three independent experiments each performed in triplicate; error bars indicate standard errors of the mean. **, *p* < 0.01.

HIF-1α induces KSHV reactivation by directly enhancing expression of its *ORF50* gene, the orthologue of EBV’s *BRLF1* gene [32,33]. Thus, we asked whether HIF-1α reactivates EBV by inducing transcription from Rp and/or Zp. HEK 293T cells were transiently co-transfected with plasmids expressing the oxygen-insensitive variant of HIF-1α, HIF-1β, and reporters driving luciferase expression from Rp or Zp. We used an Rta expression plasmid as a positive control since Rta is a potent transcriptional activator of both Zp and Rp [15]. While addition of HIF-1α/HIF-1β activated transcription from the Zp-luc reporter approximately 24-fold, it activated the Rp-luc reporter similarly to the four-fold activation observed with the negative control TATA-luc reporter (Fig 4). As expected, Rta robustly activated both reporters. Thus, HIF-1α/HIF-1β heterodimers activate transcription from Zp approximately six-fold above the non-specific level observed in this assay while failing to activate specifically transcription from Rp. Thus, in contrast to KSHV, we conclude that HIF-1α regulates lytic EBV infection by activating expression of the *BZLF1* gene, not the *BRLF1* gene.

### HIF-1α directly activates transcription from Zp via an HRE

**Fig 5:**
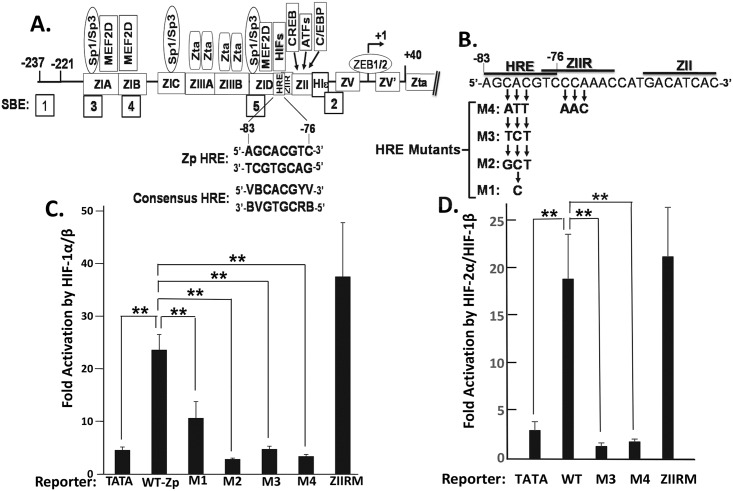
Zp contains a transcriptionally functional HRE activated by both HIF-1α and HIF-2α. (A) *In silico* identification of a consensus HRE located from nt -83 through -76 relative to the transcription initiation site of Zp. Shown here is a schematic indicating *cis*-acting regulatory elements (denoted by rectangles) along with their *trans*-acting factors where known. SBE, SMAD-binding element. (B) Schematic indicating the sequence alterations present in the base pair substitution mutants, M1-M4 and ZIIRM, analyzed here. The lines above the sequence indicate the locations of the HRE, ZIIR, and ZII elements. (C, D) Both HIF-1α- and HIF-2α-dependent activation of transcription from Zp map to the putative HRE. 293T cells maintained in 24-well plates were co-transfected with (i) 200 ng of pTATA-luc as a control, pWTZp-luc, or the base pair substitution variants of pWTZp-luc depicted in Fig. 5B, and (ii) pHA-HIF-1αP402A/P564A-pcDNA3 (panel C) or pHA-HIF-2αP405A/P531A-pcDNA3 (panel D) plus pHIF-1β (40 ng each) or 80 ng pcDNA3. Cells were harvested 48 h later, and luciferase activities were determined as described in the legend to Fig 4. Data are averages from three or more independent experiments each performed in triplicate. **, *p* < 0.01.

To determine how HIF-1α activates *BZLF1* gene expression, we performed an *in silico* analysis of Zp and noted a single consensus HRE located from nt -83 through -76 relative to the Zp transcriptional initiation site (Fig 5A). To examine whether HIF-1α-dependent transactivation of Zp mapped to this sequence, we constructed a set of base-pair substitution mutant variants of our WT luciferase reporter, pWTZp-luc (Fig 5B). These mutations were designed to avoid disrupting bases that overlap the adjacent ZIIR silencing element [34,35]. Reporter assays performed with these variants of pZp-luc showed that the WT and ZIIR mutant promoters were activated by HIF-1α/HIF-1β approximately five- to eight-fold above the non-specific activation observed with the minimal TATA box-containing control promoter while none of the 3-bp substitution mutants in the putative HRE were activated above this non-specific level (Fig 5C). Even the 1-bp substitution mutation present in mutant M1 significantly reduced activation by HIF-1α/HIF-1β. Analysis of the basal activity of these mutants in the absence of HIF-1α and of a non-overlapping mutant only altered in nt -77 and -76 of the Zp HRE ruled out the possibility that these HRE mutations were affecting binding of a repressor (S2 Fig). Similar results were obtained when we used an expression plasmid that encodes an oxygen-insensitive variant of HIF-2α in place of the HIF-1α one (Fig 5D). Thus, we conclude that Zp contains a transcriptionally functional HRE that includes nt -79 through -81.

### HIF-1α binds the Zp HRE

**Fig 6:**
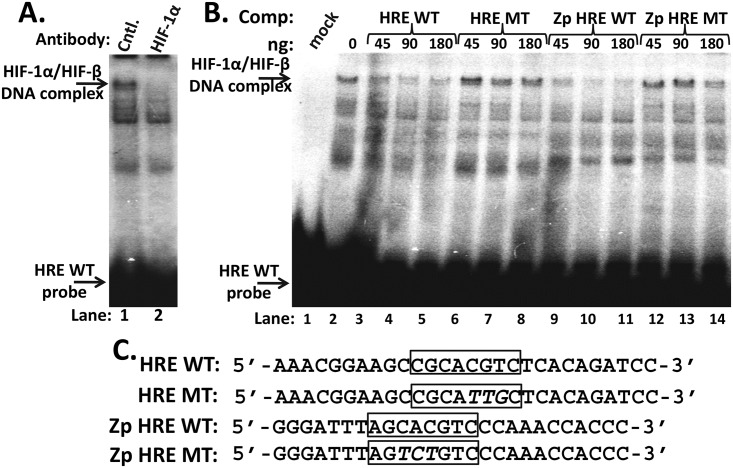
HIF-1α sequence-specifically binds the nt -80 region Zp HRE. (A) EMSA showing HIF-1α binding to a radiolabeled, double-stranded oligonucleotide that contains the consensus HRE WT sequence shown in panel C. Approximately 30 μg protein obtained from a nuclear extract prepared from CoCl_2_–treated AGS cells was pre-incubated with 1 μg anti-HIF-1α polyclonal antibody (lane 2) or 1 μg anti-IgG antibody as a control (lane 1) prior to addition of the probe DNA and electrophoresis. (B) Competition EMSA showing sequence-specific binding of HIF-1α to the Zp HRE. Assays were performed by pre-incubation of the reaction mixture with the indicated unlabeled, double-stranded competitor oligonucleotides at the indicated amounts prior to addition of the radiolabeled probe and electrophoresis. (C) Sequences of the oligonucleotides used as probe (HRE WT) and competitors. Mutated bases indicated by italicized font. Boxes, HREs.

HREs act as sequence-specific binding elements for HIF-α/β heterodimeric complexes. To demonstrate that HIF-1α/HIF-1β heterodimers bind to the Zp HRE, we performed *in vitro* DNA-binding assays. Our protein source of HIF-1α/HIF-1β complexes was nuclear extract obtained from EBV-negative AGS cells incubated for 24 h with 200 μM CoCl_2_, an iron competitor. A radiolabeled, double-stranded oligonucleotide containing a consensus HRE sequence, 5’-CACGTC-3’, served as probe (Fig 6C, HRE WT). We identified the HIF-1α-containing protein-DNA complex by showing it was lost by incubation with a HIF-1α-specific antibody (Fig 6A). Competition electrophoretic-mobility-shift assays (EMSAs) were performed by pre-incubation of the extract with various amounts of the unlabeled, double-stranded WT or mutant (MT) oligonucleotides indicated in panel C. WT Zp HRE-containing oligonucleotide competed for binding the HIF-1α/HIF-1β complex as well as the consensus WT HRE oligonucleotide (Fig 6B, lanes 9–11 vs. lanes 3–5, respectively) while the 3-bp mutant variant of this consensus HRE oligonucleotide failed to compete (Fig 6B, lanes 6–8 vs. lane 2). Likewise, a 3-bp mutant variant of the Zp HRE-containing oligonucleotide corresponding to the M3 mutation that abolished HIF-1α/HIF-1β-dependent transcriptional activation of Zp-luc (Fig 5) also largely failed to compete for binding HIF-1α/HIF-1β complexes (Fig 6B, lanes 12–14 vs. lane 2). Thus, the *trans*-activation and DNA-binding activities of HIF-1α co-localize to the HRE present within Zp.

### HIF-1α binds Zp *in vivo*

**Fig 7:**
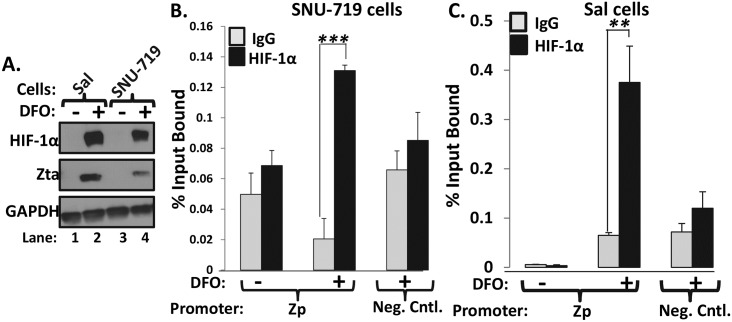
HIF-1α binds to Zp in context of whole EBV genomes. (A) Immunoblots showing relative levels of HIF-1α and Zta present in Sal and SNU-719 cells incubated for 24 h with (+) or without (-) 200 μM DFO prior to processing for ChIP analysis. (B, C) Quantitative-PCR analyses of the chromatin obtained from the cells in panel A following precipitation with anti-HIF-1α or anti-IgG antibodies. The primer pairs spanned Zp versus a sequence located 4.8-kbp upstream of Zp as a negative control (neg. cntl.). Data presented are average Ct values of two independent experiments each performed in triplicate; error bars indicate standard deviations. **, *p* < 0.01; ***, *p* < 0.001.

We next performed ChIP assays to show HIF-1α binds Zp in the physiological context of whole EBV genomes. SNU-719 and Sal cells incubated (+) or not (-) with 200 μM DFO for 24 h served as the source of chromatin given this treatment induces abundant accumulation of HIF-1α in these cells (Fig 7A). Quantitative PCR analysis of these samples following chromatin precipitation with HIF-1α-specific versus IgG control antibody indicated that this HIF-1α-specific antibody precipitated Zp approximately four-fold more efficiently than did the anti-IgG antibody (Fig 7B and 7C), yet failed to increase significantly precipitation of EBV DNA located approximately 4.8 kbps upstream of Zp (Neg. Cntl.). Thus, we conclude that the EBV *BZLF1* gene contains a transcriptionally functional, HIF-1α-binding HRE within Zp.

### HIF-1α-induced reactivation of EBV requires the Zp HRE

**Fig 8:**
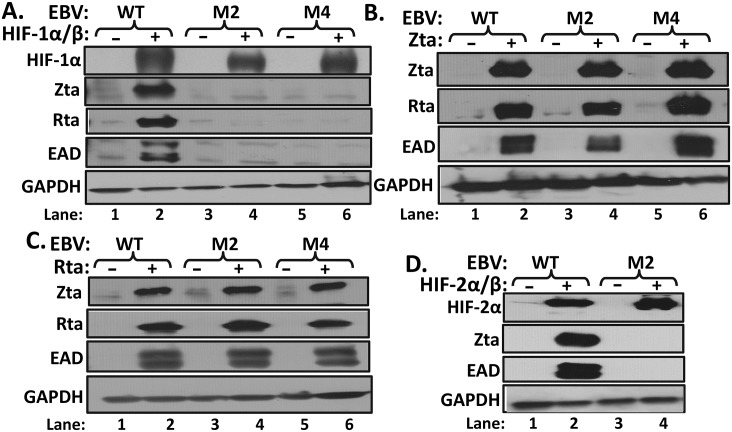
HIF-α-induced lytic reactivation of EBV requires the Zp HRE. Even-numbered lanes (+), the indicated WT- and HRE mutant-infected 293T cell lines grown in 6-well plates were transfected with: (A) 0.5 μg each of plasmids expressing HIF-1β and the oxygen-insensitive variant of HIF-1α; (B) 50 ng of the Zta-expressing plasmid, pCMV-BZLF1; (C) 100 ng of the Rta-expressing plasmid, pRTS15; or (D) 0.5 μg each of plasmids expressing HIF-1β and the oxygen-insensitive variant of HIF-2α. Whole-cell extracts were prepared 72 h later and analyzed by immunoblotting for the indicated proteins. Correspondingly similar amounts of pcDNA3 DNA were transfected in parallel in the odd-numbered lanes (-) as controls.

To confirm that HIF-1α induction of lytic EBV infection truly occurs via binding to this Zp HRE rather than indirectly via downstream signaling events, we constructed two independent HRE variants of EBV containing the M2 and M4 substitution mutations analyzed in our reporter assay (Fig 5) within the context of the p2089 BAC [36]. 293T cells were transfected in parallel with these two EBV HRE mutant BACs alongside their parental WT EBV BAC and selected for resistance to hygromycin to establish the cell lines 293T-EBV M2, 293T-EBV M4, and 293T-EBV WT, respectively. Confirming our observation with AGS-Akata cells (Fig 3A), co-transfection of 293T EBV-WT cells with plasmids expressing the oxygen-insensitive variant of HIF-1α along with HIF-1β efficiently induced expression of EBV IE and E genes (Fig 8A, lane 2 vs. lane 1). Strikingly, co-transfection of HIF-1α/HIF-1β expression plasmids into 293T cells latently infected with either the M2 or M4 HRE mutant variant of EBV failed to induce synthesis of lytic EBV antigens above the background level of spontaneous reactivation (Fig 8A, lane 4 and lane 6, respectively).

To rule out non-HRE-related causes for our negative finding, we also transfected these cell lines with plasmids expressing Zta (Fig 8B) or Rta (Fig 8C). When either of these EBV IE proteins was provided, all three cell lines exhibited similar high-level expression of both the non-transfected IE gene and the EAD-encoding gene, *BMRF1* (Fig 8B, lanes 2, 4, and 6; Fig 8C, lanes 2, 4, and 6). We also recovered the viral DNAs from these mutant-infected cell lines and thoroughly analyzed their genomes for second-site mutations; none were found by either DNA sequencing or restriction fragment pattern analysis. Thus, mutation of the Zp HRE within the context of whole EBV genomes disables HIF-1α-dependent induction of *BZLF1* gene expression. We conclude that HIF-1α induces lytic reactivation in EBV primarily (possibly, exclusively) via direct binding to this single HRE located within Zp.

Some HREs respond to both of the two major HIF-α isoforms whereas others primarily or solely respond to only one of them [18]. As indicated above, we observed that HIF-1α RNA is more abundant that HIF-2α RNA in all of the EBV^+^ epithelial and B cell lines and tumors we have examined to date. Nevertheless, given our finding that HIF-2α can also activate Zp in reporter assays (Fig 5), it remains possible that some conditions exist (*e*.*g*., chronic hypoxia) in which HIF-2α is the more physiologically important HIF-α regarding some aspects of EBV’s life cycle. Thus, we examined likewise whether latent EBV genomes can also be induced into lytic infection using a plasmid that expresses an oxygen-insensitive variant of HIF-2α. As with HIF-1α, we observed high-level induction of EBV IE and E gene expression in the cells latently infected with the WT EBV genome, but not cells latently infected with the Zp HRE M2 mutant (Fig 8D, lane 2 vs. lane 4, respectively). Noteworthy is the fact that neither HIF-1α nor HIF-2α directly activated *BRLF1* gene expression in cells infected with Zp HRE mutant genomes; if they had, we should have seen synthesis of Zta and EAD as well as Rta protein as was observed in Fig 8C. This finding demonstrates that HIF-α/β heterodimers fail to activate transcription from Rp in the context of full-length latent EBV genomes as well as in reporter assays (Fig 4). Thus, we conclude that, when present, either of the two major HIF-α isoforms can mediate EBV reactivation via the Zp HRE.

### HIF-1α protein accumulates during epithelial and B-cell differentiation

**Fig 9:**
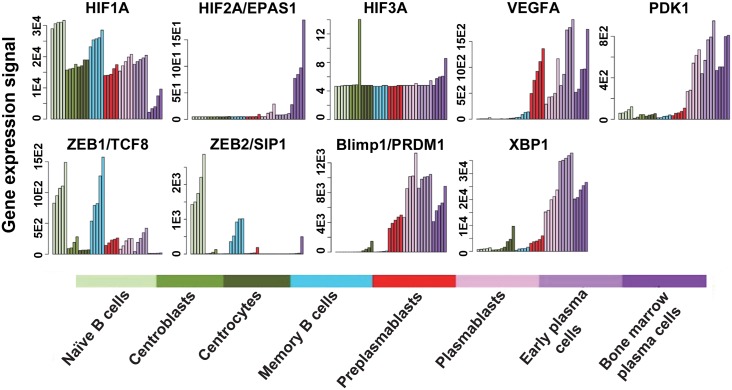
Differentiation of memory B cells into plasmablasts induces HIF-1α activity. Changes in RNA levels during differentiation of primary human B cells into plasma cells of some cellular genes (*VEGFA*, *PDK1*) whose expression is known to be activated by HIF-1α and some other genes whose products (ZEB1, ZEB2, Blimp-1α, XBP-1s) are known to contribute to regulation of *BZLF1* gene expression. Relative levels of these RNAs were determined by extraction of data from five sets of microarray analyses of mRNA purified from cells harvested at the eight indicated stages of B-cell differentiation. Note large differences in scales shown for y-axes.

Why might EBV have evolved to contain an HRE within Zp? To answer this question, we examined whether the appearance of HIF-1α protein during differentiation of normal epithelial and B cells coincides with the cell types in which lytic EBV infection takes place. In B cells, lytic EBV reactivation occurs when memory B-cells begin to differentiate into plasma cells [2]. To determine when functionally active HIF-1α protein is present in B cells, we mined existing microarray data sets obtained from B cells harvested at eight different stages of differentiation, ranging from naïve B cells to fully differentiated plasma cells (Fig 9). HIF-1α mRNA is present at high levels in all of these stages, declining somewhat only during the very last stage. However, functionally active HIF-1α protein, as measured by expression of the HIF-1α-activated genes *VEGFA* and *PDK1*, dramatically increases in the post-memory cell preplasmablast and plasmablast stages, respectively. These are the same stages during B-cell differentiation when expression of both ZEBs plummets (possibly due, in part, to HIF-1α also activating synthesis of miR-429 [37], a down-regulator of ZEB levels [38,39]), and expression of Blimp-1α and XBP-1 dramatically increases. Thus, the stages during B-cell differentiation when EBV reactivates are coincident with the stages when three of the Zp activators (HIF-1α, Blimp-1α [40], and XBP-1s [41,42]) appear and two of the major Zp repressors (ZEB1 and ZEB2 [43,44]) disappear.

**Fig 10:**
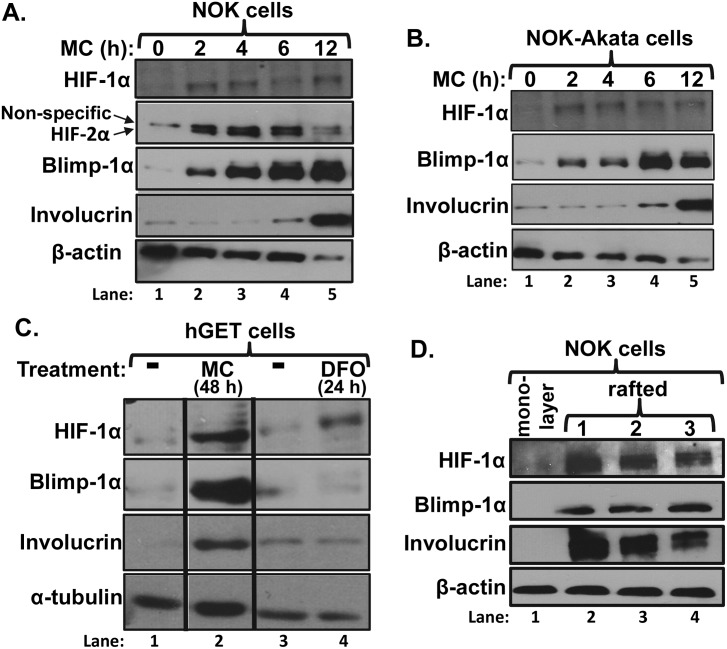
Differentiation of epithelial cells induces accumulation of HIF-1α. Immunoblots showing changes with time in HIF-1α protein levels following induction of differentiation of (A) NOK and (B) NOK-Akata cells by suspension in methylcellulose (1.6% in K-SFM) for the times indicated. (C) Immunoblots showing HIF-1α accumulation in hGET cells following induction of differentiation by suspension in methyl cellulose (1.6% in K-SFM for 48 h; lane 2). As controls, the cells for lanes 1, 3, and 4 were incubated in parallel in K-SFM without MC. For lane 4, 50 μM DFO was added to the cells 24 h prior to harvest as a control for HIF-1α stabilization in the absence of MC-induced differentiation. (D) Immunoblots showing HIF-1α accumulation in NOK (clone #3) cells following induction of differentiation by growth in organotypic culture for 11 days at an air-liquid interface. Lanes 2–4 contain protein extracts from three different rafts. Lane 1 contains protein extract from NOK (clone #1) cells maintained in an undifferentiated state as a monolayer grown in K-SFM. Whole-cell extracts were prepared and analyzed for the indicated proteins.

Another stage of EBV’s natural life cycle involves the infection of differentiated epithelial cells by EBV (either free virions or virus produced in reactivated EBV^+^ B cells) [10]. Expression of Blimp-1α is also induced during epithelial cell differentiation, synergizing with KLF4 to activate transcription from both Zp and Rp [40,45]. To determine whether HIF-α protein accumulation is induced by epithelial cell differentiation, we incubated telomerase (TERT)-immortalized human normal oral keratinocyte (NOK) cells with the differentiation-inducing agent, methylcellulose (MC) (Fig 10A). Both HIF-1α and HIF-2α protein, along with some Blimp-1α, appeared within 2 h of MC addition; they remained present for at least 12 h. Thus, their stabilization may be among the earliest events to occur during epithelial cell differentiation, hours before the appearance of involucrin, another marker of epithelial cell differentiation. The kinetics of appearance of HIF-1α and Blimp-1α were similar in MC-treated NOK-Akata, cells infected with EBV (Fig 10B). This latter finding suggests that regulation of the stabilization of HIF-1α protein during epithelial cell differentiation occurs independently of the presence of EBV. We examined likewise hTERT-infected human gingival epithelial (hGET) cells. In this case, HIF-1α protein and Blimp-1α were both abundantly present, along with involucrin, 48 h after addition of MC (Fig 10C, lane 2); accumulation of HIF-1α protein was within a few-fold of that observed when these cells were incubated with 50 μM DFO (Fig 10C, lane 4). HIF-1α protein also accumulated together with Blimp-1α and involucrin when NOK cells were induced to differentiate by growth in organotypic culture (Fig 10D). Thus, both HIF-1α and Blimp-1α are present in differentiated cells of the types present in the human oral cavity.

Thus, we conclude that HIF-1α protein accumulates during the course of both epithelial and B-cell differentiation, likely contributing to activation of *BZLF1* gene expression along with other inducers of Zp activation.

### HIF-1α also enhances lytic EBV reactivation *in vivo*

**Fig 11:**
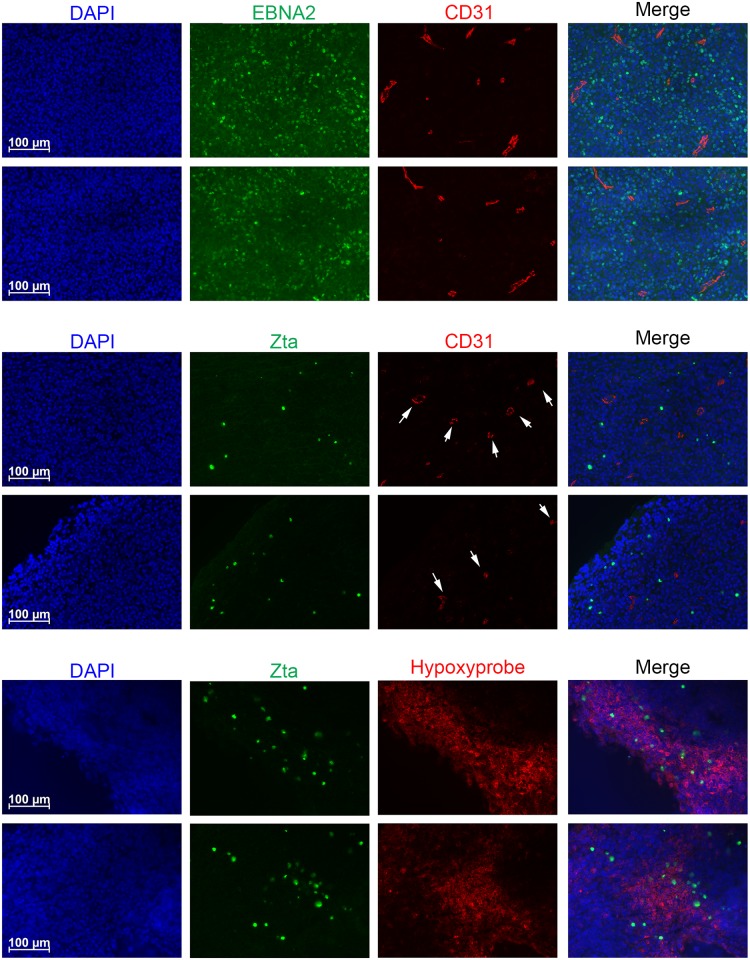
Most Zta^+^ cells present in B-cell lymphomas induced by EBV in mice with a humanized immune system reside distal to blood vessels. NSG mice were inoculated i.p. with human cord blood that had been infected 1.5 h earlier with the M81 strain of EBV. Thirty-three days later, the mice were sacrificed, and the tumors were flash frozen, sectioned, and processed by IFS for the indicated proteins. (A) Sections co-stained for EBNA2 (green) and CD31 (red). (B) Sections co-stained for Zta (green) and CD31 (red). (C) Sections co-stained for Zta (green) and Hypoxyprobe (red). All sections were counterstained with DAPI (blue). These sections are representative of data observed with over two dozen EBV^+^ tumors obtained in several experiments performed with blood from different donors. Arrows indicate locations of some of the blood vessels based upon cross-reactivation with CD31 antibody.

Given the above findings, we hypothesized that hypoxic regions within growing EBV^+^ tumors accumulate HIF-1α, thereby increasing the probability that latent EBV infection will reactivate into lytic replication. To test this hypothesis, we examined EBV^+^ B-cell lymphomas [similar in phenotype to human diffuse large B-cell lymphomas (DLBCLs)] that had been induced in NSG (NOD/LtSz-*scid/IL2Rγ*^*null*^) mice by inoculation with human cord blood that had been infected with the M81 strain of EBV by co-culture for 90 min immediately prior to injection [46]. If our hypothesis is valid, EBV^+^ tumor cells located distally from blood vessels (*i*.*e*., in poorly oxygenated regions) are more likely to reactivate into lytic replication than ones located near them. Latently EBV-infected B-cells were identified by IFS for the latent EBV protein, EBNA2 (Fig 11A); lytic infection was identified by IFS for the lytic EBV protein, Zta (Fig 11B and 11C; see also S3 Fig for adjacent serial sections, including H&E staining). The blood vessels were identified by co-staining with a CD31 (PECAM-1)-specific antibody that detects endothelial cells. Hypoxic regions were identified by co-staining with a Hypoxyprobe-specific antibody in mice that had been treated with Hypoxyprobe 90 min prior to sacrifice. Strikingly, whereas the EBV^+^ EBNA2 cells were located throughout these tumor sections, as expected, Zta^+^ cells were only occasionally observed within three cell widths of blood vessels.

**Fig 12:**
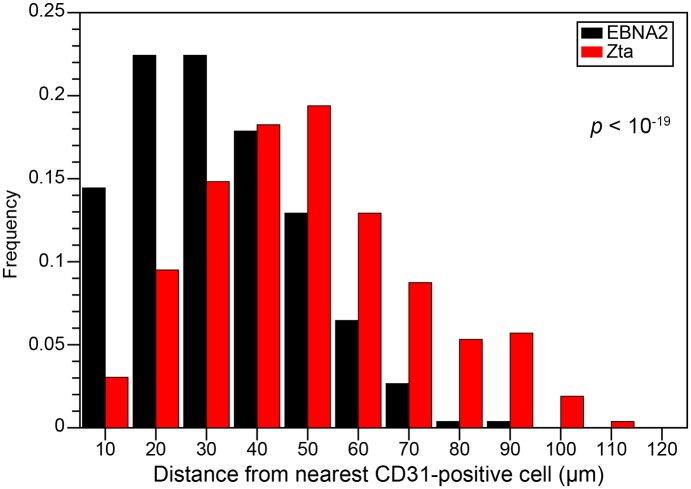
Histogram showing distributions of distances of EBNA2^+^ versus Zta^+^ cells from the nearest blood vessel (*i*.*e*., a CD31^+^ cell). Distances for 263 EBNA2^+^ and 263 Zta^+^ cells were measured from stained sections similar to the ones shown in Fig 11 and S3 Fig.

The distributions of distances of EBNA2^+^ versus Zta^+^ cells from their nearest blood vessel are summarized in Fig 12: while most EBNA2^+^ cells were located within 30 μm of a blood vessel, most Zta^+^ cells were located beyond this distance (*p* < 10^−19^). Dual staining for Zta and Hypoxyprobe pictorially documented that most of the Zta+ cells were located within or near hypoxic regions of the tumors, distal to blood vessels (Fig 11C, S3 Fig). IFS of xenografts generated by injection of gastric cancer-derived SNU-719 cells into the flanks of NSG mice (*e*.*g*., S4 Fig) and IHC staining of serial sections of M81-induced tumors (*e*.*g*., S5 Fig) produced similar findings. Thus, we conclude that the probability of a latently infected cell reactivating *in vivo* is considerably higher when it is located in an oxygen-deficient environment.

## Discussion

This study is the first one to identify the mechanism by which hypoxia induces lytic EBV infection, leading us to conclude that HIF-1α plays roles in both EBV’s natural life cycle and EBV-associated tumorigenesis. We demonstrated with shRNA knockdown experiments that HIF-1α was required for induction of EBV reactivation by the hypoxia mimic DFO (Fig 3B). Expression of an oxygen-insensitive variant of HIF-1α was also sufficient to induce EBV reactivation (Figs 3A and 8A). HIF-1α activated transcription from Zp, but not Rp, in both a reporter assay and in the context of whole EBV genomes (Figs 4 and 8A), with activation mediated by a consensus HRE located at nt -83 through -76 relative to the transcription initiation site of Zp (Figs 5 and 8). EMSAs and ChIP assays confirmed that HIF-1α bound Zp via this HRE (Figs 6 and 7). Remarkably, 3-bp substitution mutations in this HRE were sufficient to eliminate HIF-1α-mediated reactivation in the context of the intact viral genome (Fig 8). Thus, HIF-1α induces lytic EBV infection by sequence-specific binding to a single HRE located within Zp.

Consistent with HIF-1α playing roles in EBV’s natural life cycle, we showed that the stages during epithelial and B-cell differentiation during which HIF-1α and Blimp-1α are present correlate with the onset of lytic EBV infection (Figs 9 and 10). We also presented data from EBV-induced B-cell lymphomas and gastric cancer xenografts indicating that hypoxia increases the frequency of lytic EBV reactivation *in vivo* (Figs 11 and 12; S3–S5 Figs). Thus, HIF-1α likely also contributes to EBV-associated tumorigenesis given a small amount of lytic infection is known to enhance tumor growth (reviewed in [47]).

Lastly, we used several compounds that induce stabilization of HIF-αs to investigate how hypoxia triggers lytic EBV infection. We showed that incubation with deferoxamine, an FDA-approved drug with long-standing clinical uses, promoted accumulation of HIF-1α and lytic EBV antigens in EBV^+^ cells of both epithelial and lymphocytic origin (Fig 1). A 24-h treatment with DFO was sufficient to induce high-level synthesis of early and late lytic EBV antigens by 48 h (Fig 1C). L-mimosine, another iron-chelating drug, also efficiently induced synthesis of Zta (Fig 1A), but was not investigated further because its clinical use is restricted to topical applications due to toxicity. MLN4924, a drug currently in phase I/II clinical trials for a variety of cancers, induces EBV reactivation at least as well as DFO (Fig 1B). Thus, we conclude that HIF-α-stabilizing drugs may have utility in lytic-induction therapy for treating patients with a variety of EBV-associated cancers.

### HRE versus ZIIR elements of Zp

The HRE identified here overlaps the previously identified ZIIR element of Zp [34,35] (Fig 5B). Thus, one possibility was that binding of HIF-1α to this HRE activates transcription from Zp by displacing the yet-to-be-identified ZIIR repressor. Inconsistent with this hypothesis was our finding that mutations known to relieve ZIIR-mediated repression affected neither HIF-1α- nor HIF-2α-induced activation of transcription from Zp (Fig 5C and 5D, respectively) unless they also impinged upon the HRE element (S2 Fig). Furthermore, HRE mutations that abolished HIF-α-induced reactivation of EBV had no effect on the frequency of spontaneous reactivation (Fig 8), a frequency enhanced in ZIIR mutant variants of EBV [35]. Thus, we conclude that the HRE and ZIIR elements are genetically distinguishable, independently acting regulatory elements of Zp, with HIF-α proteins functioning as transcriptional activators via binding to the HRE.

### HIF-1α versus HIF-2α

The sequence encompassing the HRE present in the promoter of KSHV’s latent gene, *ORF73* [encoding latency-associated nuclear antigen (LANA)], is identical to that of the EBV Zp HRE we identified here, with both HREs being responsive to both major HIF-α isoforms [48] (Figs 5 and 8). However, HIF-1α was the predominant HIF-α expressed at the RNA level in all of the EBV^+^ primary tumors and cell lines we have examined to date. Consistent with this finding, the gastric cancer-derived cell lines, SNU-719 and AGS-Akata, were the only ones in which we detected HIF-2α protein upon incubation with DFO (Fig 1D). Previous reports of others likewise indicated preferential accumulation of HIF-1α protein with exposure to hypoxia in EBV^+^ LCLs that contain little HIF-2α mRNA [49] and in EBV^+^ NPC-derived cell lines that contain some HIF-2α mRNA [50]. Thus, we conclude that HIF-1α is the primary HIF-α of physiological relevance to EBV’s natural life cycle and in EBV^+^ tumors.

### Role of HIF-1α in EBV’s natural life cycle and tumorigenesis

Much literature exists indicating HIF-1α plays central roles in regulating both lytic infection and tumorigenesis by KSHV (reviewed in [22,23]). Functional HREs are present within the promoter regions of KSHV’s latent gene, *ORF73/LANA*, as well as its IE lytic gene, *ORF50/RTA*, and lytic *ORF34-ORF37* gene cluster [32,48,51]. HIF-1α complexes with LANA to activate *ORF50* gene expression during hypoxia, inducing lytic KSHV replication [33], yet a SUMOylated form of LANA inhibits HIF-1α induction of RTA synthesis to maintain latency during normoxia while still enabling HIF-1α to promote angiogenesis [52].

As with KSHV, the relationship between EBV’s latent gene products and HIF-1α is also complex. EBNA3A and EBNA-LP bind PHDs, blocking their catalytic activity and, thereby, inhibiting oxygen-dependent degradation of HIF-1α [53]. LMP1 promotes accumulation of HIF-1α by signaling PHD1 and PHD2 degradation pathways [54,55]. EBNA3A stabilizes HIF-1α via protein-protein interactions [53], a complex somewhat analogous to the LANA/HIF-1α complex. However, these above-mentioned EBV-encoded proteins are clearly not necessary for HIF-1α-induced activation of *BZLF1* gene expression given we showed here that HIF-1α can induce transcription from Zp in EBV-negative cells and Zta synthesis in EBV^+^ Sal cells that are in a Wp-restricted latency in which these proteins are not expressed. In latency types in which these above-mentioned proteins stabilize HIF-1α, other factors are likely also present in the cells to inhibit HIF-1α from inducing lytic reactivation. EBNA1, present in all EBV^+^ cells, also has been reported to enhance HIF-1α activity, most likely indirectly via its effects on AP-1 [56].

**Fig 13:**
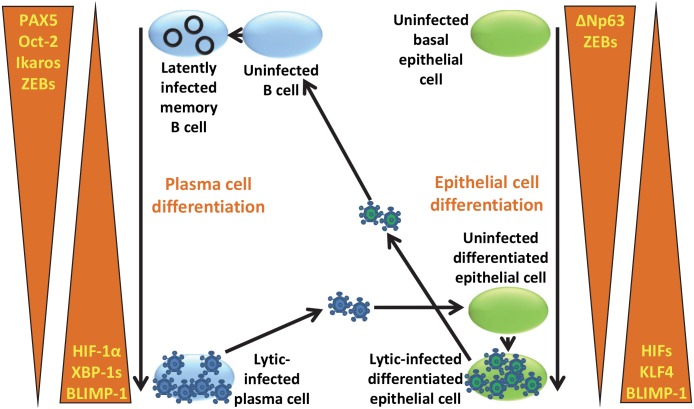
Model for how differentiation of epithelial and B cells may induce reactivation of EBV into lytic infection and influence ability to be infected by EBV via altering levels of cellular transcription factors known to contribute to repression and activation of *BZLF1* and *BRLF1* gene expression. See text for details.

We propose that HIF-1α plays central roles in regulating both lytic replication and tumorigenesis by EBV. Regarding EBV’s natural life cycle, we hypothesize that B cells from the naïve B-cell through memory B-cell stages lack functional HIF-α activity as well as Blimp-1α and XBP-1s (Fig 9) (other known inducers of *BZLF1* gene expression), while containing several known direct or indirect repressors of this gene [9,43] and inhibitors of Zta activity [57,58] (Fig 13). Thus, EBV infection tends to go latent. However, if an EBV-infected B cell begins to undergo plasma cell differentiation, the virus may reactivate due to the appearance of functionally active HIF-1α along with these other activators and loss of these repressors. Likewise, when epithelial cells differentiate, they accumulate HIF-αs along with Blimp-1α and KLF4 (known inducers of *BRLF1* as well as *BZFL1* gene expression [40,45]) and lose repressors of these genes such as the ZEBs (Fig 13). Thus, when EBV virion particles or EBV-infected B cells come into close contact with differentiated epithelial cells within the oral cavity, the introduction of EBV genomes into these cells can lead to lytic replication and production of infectious virus, helping to spread the virus from cell-to-cell and host-to-host.

How might HIF-1α and the Zp HRE contribute to tumorigenesis by EBV *in vivo*? We propose that HIF-αs contribute via two routes to tumor growth in EBV^+^ cancers. As is true of most tumors, EBV^+^ tumors develop hypoxic regions as they enlarge (*e*.*g*., Fig 11C, S3A Fig; [59–61]), leading to accumulation of the HIF-αs whose genes are being expressed. These HIF-αs then activate expression of a variety of cellular genes involved in angiogenesis and anaerobic metabolism that help the tumor to continue to enlarge (reviewed in [62]). In the case of EBV^+^ tumors, presence of HIF-α also contributes to activation of *BZLF1* gene expression, leading to EBV lytic-gene expression in some tumor cells. We showed here by examining EBV-induced lymphomas and EBV^+^ gastric cancer xenografts that Zta^+^ cells were preferentially located in regions of the tumors that were clearly hypoxic as indicated by Hypoxyprobe staining or, presumably, hypoxic because they were located distal to blood vessels (Figs 11 and 12, S3–S5 Figs). Thus, we propose the following model: Hypoxic regions develop in EBV^+^ tumors as they grow in size, leading to accumulation of HIF-1α and, in some cases, HIF-2α. Prior to angiogenesis, HIF-α increases the frequency of lytic EBV infection in these hypoxic regions, with these lytic-infected cells secreting a variety of cellular and viral factors, some of which contribute to the enhancement of tumor growth (reviewed in [47]).

### HIF-1α and oncolytic therapy

The goal of chemotherapy is to kill cancer cells while minimizing harm to healthy cells. Treatment of some EBV^+^ cancers with minimally toxic drugs that rapidly and efficiently induce EBV lytic-gene expression, in combination with prodrugs such as ganciclovir (GCV), may be one way to achieve this goal ([16,63] and references cited therein). Based upon the findings presented here, we propose that briefly targeting the PHDs or other enzymes that regulate degradation of HIF-1α (*e*.*g*., NAE) may be useful as part of a strategy to achieve efficient EBV lytic-induction therapy. Transient expression of HIF-1α induced sufficient Zta synthesis to promote expression of EBV early- and late-lytic genes (Fig 1C). Furthermore, these expressed early-lytic genes included *BGLF4* [as indicated by the presence of phosphorylated forms of EAD (*e*.*g*., Figs 3 and 8)], the gene that encodes the EBV-PK that can phosphorylate GCV [64]. Intrinsic features of EBV and HIF-1α make this strategy feasible: (i) Once *BZLF1* gene expression is activated by an inducer, Zta synthesis usually continues after the inducer is removed because of its positive feedback loop with Rta (*e*.*g*., Fig 1C); and (ii) The HIF-αs are rapidly degraded once HIF-α-stabilizing drugs are removed (*e*.*g*., Fig 1C). Thus, brief treatment with a stabilizer of HIF-1α may well be sufficient, reducing potential adverse reactions due to off-target effects of the drug and HIF-1α. Quite likely, one may be able to increase considerably the percentage of the EBV^+^ cells reactivated by using DFO, MLN4924, or another HIF-1α stabilizer in combination with other drugs known to activate *BZLF1* gene expression via different cellular signaling pathways (*e*.*g*., HDAC inhibitors).

We were fortunate to identify here an already FDA-approved drug as a possible candidate for use in lytic-induction therapy. DFO and the FDA-approved oral iron-chelators deferasirox and deferiprone, are used to treat iron overload and toxicity that result from frequent blood transfusions [65]. DFO-based therapy is also emerging as a tool for treating a variety of diseases, including persistent anemia, impaired angiogenesis resulting from diabetes mellitus, and numerous neurodegenerative disorders (reviewed in [66]).

## Materials and methods

### Ethics statement

The mouse experiments were approved by the UW-Madison Institutional Animal Care and Use Committee (protocol #M005197-A01) and conducted in accordance with the *NIH Guide for the Care and Use of Laboratory Animals*. The mice were sacrificed by cervical dislocation under isoflurane anesthesia. The UW IRB classified the work with human tissues and cells as exempt.

### Cells

Sal cells (a gift from Alan Rickinson via Bill Sugden) were derived from an EBV^+^ BL; they are co-infected with wild-type and EBNA2-deleted EBV genomes and maintain a Wp-restricted latency [67]. KemIII cells (a gift from Alan Rickinson via Jeff Sample), derived from an EBV^+^ BL, are currently in type III latency and express LMP1. These B-cell lines were maintained in RPMI-1640 medium supplemented with 10% FBS and 100 units/ml penicillin and 100 μg/ml streptomycin (pen-strep; Life Technologies). SNU-719 cells (obtain from Jin-Pok Kim via Bill Sugden), derived from an EBV^+^ gastric carcinoma, retain their original EBV genome [68]; they were maintained likewise. AGS-Akata cells, an EBV-infected clonal derivative of AGS cells (derived from a human gastric carcinoma; obtained from ATCC) [69], were maintained in F12 medium (Life Technologies) supplemented with 10% fetal bovine serum (FBS; Atlanta Biologicals) and pen-strep additionally supplemented with 400 μg/ml of G418.

293T (obtained from ATCC) is a human embryonic kidney (HEK) cell line expressing the early genes from SV40 and adenovirus. These cells were maintained in DMEM (Life Technologies) supplemented with 10% FBS and pen-strep. 293T cells harboring the B98.5 strain of EBV in BAC p2089 [36] or HRE mutant variants thereof were maintained in DMEM additionally supplemented with 100 μg/ml hygromycin B.

NOK (a gift from Karl Munger) are telomerase (hTERT)-immortalized normal oral keratinocyte (NOK) cells [13]. NOK-Akata, clone 2 (generously obtained from Bill Sugden), are NOK cells (with WT p53) that are latently infected with an Akata-GFP strain of EBV [13]. NOK (clones #1 and #3) cells are clonal isolates of NOK cells (with WT p53). These cell lines were maintained in an undifferentiated state by growth in keratinocyte serum-free medium (K-SFM; Life Technologies) supplemented with epidermal growth factor, bovine pituitary extract, pen-strep. The NOK-Akata growth medium also included 50 μg/ml G418.

hTERT-transduced human gingival epithelial (hGET) cells were generated as follows. A frozen pool of primary human gingival epithelial cells (HGEPp) was obtained from CellnTEC. Upon thawing, the cells were initially grown in their specialty medium (CnT-PR; CELLnTEC) and re-frozen. These cells were then passaged in K-SFM supplemented with a ROCK inhibitor (10 μM Y-27632 Di-HCl; Selleck Chemical #50-863-6) and infected with pBABE-puro-hTERT (Addgene plasmid #1771; a gift from Bob Weinberg) [70], a recombinant retrovirus expressing human telomerase. The hTERT-transduced cells were selected by incubation with puromycin (1 μg/ml), pooled, and subsequently maintained in K-SFM. All cells were incubated at 37°C in a humidified 5% CO_2_ atmosphere.

### Plasmids

Plasmids pHA-HIF-1α P402A/P564A-pcDNA3 and pHA-HIF-2α P405A/P531A-pcDNA3 express oxygen-insensitive variants of HIF-1α and HIF-2α, respectively [71]; they were obtained from William Kaelin via Addgene (#18955 and #18956, respectively). A HIF-1β expression plasmid, pSV-Sport-ARNT [72], was obtained from Christopher Bradfield. Plasmid pZpWT-luc contains the nt -221 to + 30 region of Zp relative to the transcription initiation site cloned between the *Kpn*I and *Hin*dIII sites of the luciferase reporter plasmid, pGL3-Basic (Promega) [73]. The mutant variants of it shown in Fig 5B contain the indicated base pair substitution mutations; they were generated by Quick Change methodology (Stratagene), with pZpWT-luc serving as template and synthetic oligonucleotides containing the desired mutations surrounded by 10 bases of wild-type sequence serving as primers. Plasmid pWTRp-luc contains the nt -1069 to +38 region of Rp relative to the transcriptional initiation site cloned between the *Kpn*I and *Hin*dIII sites of pGL3-Basic. Plasmid pTATA-luc [74] served as a negative control. Plasmids pSG5-BZLF1 and pRTS15 (kindly provided by Diane Hayward) express Zta and Rta, respectively, from the SV40 early promoter [75]. Plasmid pcDNA3-BRLF1 expresses Rta from the CMV IE promoter [76]. Plasmid p2089 (a generous gift from Wolfgang Hammerschmidt) is a BAC containing the entire genome of the B95.8 strain of EBV [36]. 293 cells infected with the M81 strain of EBV in a BAC were a generous gift from Henri-Jacques Delecluse [77].

### Chemical mimics of hypoxia

To mimic hypoxia, cells were incubated with the indicated concentrations of CoCl_2_, Deferoxamine (DFO, Sigma; also called Desferrioxamine, Desferal; stock solution prepared in PBS), L-Mimosine (Mim; Sigma; stock solution prepared in 10% NaHCO_3_), or MLN4924 (Pevonedistat; AdooQ Bioscience #A11260; stock solution prepared in DMSO) for the indicated time periods.

### Immunoblot analysis

Whole-cell extracts (WCE) were prepared in SUMO lysis buffer [150 mM sodium chloride, 1% Nonidet P-40, 0.5% sodium deoxycholate, 0.1% sodium dodecyl sulfate, 50 mM Tris (pH 8.0), 50 mM sodium fluoride, 50 mM β-glycerophosphate, 2 mM sodium vanadate, 1x Complete Protease Inhibitor (Roche)]. Proteins were separated by electrophoresis in SDS gels containing 4–20% (NuSep) or 10% (Biorad) polyacrylamide and transferred to nitrocellulose membranes (ISC Biosystem). After blocking by incubation for 1 h with 5% casein in TBST [10 mM Tris-HCl (pH 7.4), 0.15M NaCl, 0.1% Tween 20], the membranes were incubated overnight at 4°C in 5% casein-TBST containing antibody specific to Zta (BZLF1, 1:250, #sc-53904; Santa Cruz), Rta (BRLF1, 1:250, #11–008; Argene), EAD (BMRF1, 1:250, #VP-E608; Vector Laboratories), or VCA/p18 (BFRF3, 1:1000, #J125; East Coast Biologics) protein. Afterward, the membranes were washed, incubated for 1 h in 5% casein-TBST containing the appropriate secondary antibody (1:5000, horseradish peroxidase (HRP)-conjugated goat anti-mouse IgG, #G-21040, Thermo Scientific; 1:5000 HRP-conjugated donkey anti-rabbit IgG, #NA-934, GE Healthcare; or 1:5000, HRP-conjugated donkey anti-goat IgG, #sc-2056, Santa Cruz) washed again with TBST, incubated for 2 min in enhanced chemiluminescence (ECL) (Luminata Crescendo, #WBLUE0100; Millipore), and exposed to X-ray film (Kodak or GeneMate). To detect HIF-1α, membranes previously probed for lytic EBV antigens were washed for 1 h in TBST, incubated overnight at 4°C in 5% casein-TBST containing anti-HIF-1α polyclonal antibody (1:500 or 1:1000, ab103063; Abcam), washed again, incubated for 1 h in 5% casein-TBST containing the secondary antibody (1:5000, horseradish peroxidase-conjugated donkey anti-rabbit IgG, #NA9340V; GE Healthcare), and processed as described above. HIF-2α was detected likewise with an anti-HIF-2α antibody (1:500 or 1:1000, ab13704; Abcam) using separate membranes from the ones used for HIF-1α. In some experiments, membranes were probed likewise with anti-Blimp-1α (1:1,000, #9115; Cell Signaling) and anti-involucrin (1:3000, #I9018; Sigma) antibodies. As a loading control, membranes were also probed with anti-GAPDH (1:5000, #A00192-40; GenScript), anti-β-actin (1:15,000, #A5441; Sigma), or anti-α-tubulin (1:2,000, #T5168; Sigma) antibody as indicated.

### IFS and IHC assays

For IFS of cells grown in culture, cells were seeded onto cover slips placed within 10-cm dishes, incubated in medium with or without the indicated concentration of DFO for 24 h, and fixed by incubation with cold methanol:acetone (1:1) for 10 min immediately after washing with cold PBS containing DFO or after incubation in medium without DFO for the indicated additional times. Non-specific antibody binding was blocked by incubation with Blotto [5% casein, 5% donkey serum (Sigma)] for 2 h at room temperature. Cells were probed for Zta protein by incubation at 4°C overnight with mouse anti-BZLF1 antibody (1:300 in Blotto, #sc-53904; Santa Cruz) followed by incubation for 2 h at room temperature with secondary antibody conjugated to a fluorescent dye (1:500, donkey anti-mouse IgG with Alexa Fluor 488, #37114; Invitrogen). After washing, DNA was stained by incubation with 4’, 6-diamidino-2-phenylindole (DAPI, 1:2,000), for 15 min at room temperature. Cells were stained likewise for HIF-1α by primary incubation with rabbit anti-HIF-1α antibody (1:500, #GTX127309; GeneTex) followed by incubation with Alexa Fluor 647 anti-rabbit secondary antibody (1:500, #A32733; Molecular Probes).

Frozen M81 and SNU-719 tumor sections (8 μm and 10 μm, respectively) were fixed for IFS in cold acetone (for Hypoxyprobe and Zta co-stain) or -20°C methanol (for EBNA2, Zta, and CD31 co-stains), and blocked in PBS with 0.1% Tween-20 and 5% goat serum (EBNA2 co-stain with CD31), 5% casein, 5% donkey serum (Zta co-stain with Hypoxyprobe), or 5% casein, 5% goat serum (Zta co-stain with CD31). Sections were then incubated in the indicated primary antibody overnight. The antibodies used were as follows: anti-Zta primary (1:100, BZ1; Santa Cruz) or anti-EBNA2 primary (1:50, #ab90543; Abcam), followed by goat anti-mouse IgG with Alexa Fluor 488 (1:250, #A11001; Invitrogen); anti-CD31/PECAM1 primary (1:50, #ab28364; Abcam) followed by goat anti-rabbit IgG with Alexa Fluor 594 (1:500, #A11012; Invitrogen); and Hypoxyprobe primary (1:50, #PAb2627AP; Hypoxyprobe, Inc.) followed by donkey anti-rabbit with Alexa Fluor 594 (1:500, #A21207; Invitrogen). Images were taken and distance measurements were determined with a Zeiss AxioImager M2 microscope and Axiovision Software version 4.8.2.

For the IHC studies (S1 and S5 Figs), the cells and M81-induced lymphomas were fixed immediately after harvest, embedded in paraffin, sectioned, deparaffinized, the antigens retrieved by incubation with 10 mM citrate buffer (pH 6.0) containing 0.05% Tween 20 for 20 min at 98°C, and processed as previously described [46,78,79]. Sections were probed for the indicated proteins using the following antibodies: CD20 (1:600, clone H1; BD Biosciences); EBNA2 (1:100, PE2; Leica Microsystems); and Zta (1:200, BZ1; Santa Cruz).

### Transient transfections

For reporter assays, 293T cells maintained in 24-well plates were co-transfected using TransIT-LT1 (Mirus Corp.) with (i) 45 ng pHA-HIF-1α P402A/P564A-pcDNA3 plus 45 ng pHIF-1β or 45 ng of each of their parental expression plasmids as controls, and (ii) 200 ng of the indicated luciferase reporter plasmid. Cells were harvested 24-to-48 h later, lysed with Passive Lysis Buffer (Promega), and luciferase activity was determined according to the manufacturer’s instructions. All assays were performed in triplicate on three or more occasions. For all other assays, expression plasmids were transfected into the indicated cells using TransIT-LT1 and the amounts of DNA indicated followed by incubation at 37°C for the times indicated prior to harvesting and processing as indicated in each figure legend.

### Knockdown of HIF-1α

AGS-Akata cells maintained in 10-cm dishes were transiently transfected when approximately 60% confluent using TransIT-LT1 with 0.8 μg each of five pLKO.1-based lentiviral vector DNAs encoding shRNAs that target HIF-1α (plasmids #3808, #3809, #3810, #3811, and #10819; Thermo Scientific). As controls, cells were transfected with 4 μg of pLKO.1 expressing the non-targeting shRNA 1864 (cntl. #1, #1864; Addgene) or NT (cntl. #2, #SHC002; Sigma-Aldrich). Two days later, cells were incubated with 200 μM DFO for 24 h, harvested, lysed in SUMO buffer, and processed for immunoblot analysis.

To transduce Sal cells with these shRNA-encoding lentiviruses, the lentiviruses were first packaged into virions as described by Open Biosystems. 293T cells in 10-cm-diameter dishes were co-transfected with (i) 0.8 μg of the five individual shRNA lentiviral vectors targeting HIF-1α or 4 μg of non-targeting shRNA cntl. #1 lentiviral vector, (ii) 1.4 μg of pCMV-dR8.2 dvpr (#8455; Addgene), and (iii) 0.6 μg of a plasmid encoding vesicular stomatitis virus G protein (VSV-G) (gift from Bill Sugden). The medium containing the virus was harvested 72 h later, passed through 0.8-μm-pore-size filters, and used to infect the Sal cells subsequently processed as described above for AGS-Akata cells except that the DFO was added three days after infection with the lentiviruses.

### Electrophoretic-mobility-shift assays

The protein source was nuclear extract prepared as previously described [40] from AGS cells that had been incubated with 200 μM CoCl_2_ for 24 h. The probe was the 5’-end-labeled, double-stranded oligonucleotide, 5’- AAACGCAAGCCGCACGTCTCACAGATCC-3’ (underlined sequence indicates consensus HRE). Reactions were performed with 20 mM HEPES (pH 7.9), 0.1 M KCl, 6 mM MgCl_2_, 4 μg poly(dI-dC):(dI-dC), 0.5 mM PMSF, 0.5 mM DTT, 8% Ficoll in a final volume of 20 μl. For immunoshift EMSAs, 10–100 μg of protein extract was pre-incubated in the reaction buffer for 20 min at 4°C with 1 μg anti-HIF-1α polyclonal antibody (#ab103063, Abcam) prior to addition of the radiolabeled probe and incubation at room temperature for 15 min. For competition EMSAs, unlabeled, competitor double-stranded oligonucleotides were pre-incubated likewise with the reaction mixture prior to addition of the radiolabeled probe. Protein-DNA complexes were separated by electrophoresis at 200 V for 2 h at 4°C in a non-denaturing 4% polyacrylamide gel with 0.5X Tris-borate-EDTA (TBE) as the running buffer. Gels were dried and imaged on a STORM 840 phosphorimager (GE Healthcare).

### Chromatin immunoprecipitation assays

Chip assays were performed essentially as previously described [40] using approximately 2 x 10^7^ SNU-719 and Sal cells grown in 15-cm dishes. Cells were incubated for 24 h with 200 μM DFO (+) or PBS (-) in medium as indicated. Protein-DNA complexes were cross-linked by incubation with 1% formaldehyde for 10 min at room temperature. Cross-linking was quenched by addition of glycine to 0.125 M. Cells were harvested by centrifugation and snap frozen until lysed. Following lysis, nuclei were isolated by centrifugation, and chromatin was sheared by sonication to approximately 500-bp size. After centrifugation to pellet debris, chromatin was divided into aliquots incubated overnight at 4°C with 2 μg of mouse anti-HIF-1α (#ab8366; Abcam) or anti-IgG (#sc-2025; Santa Cruz) antibody as a negative control. Antibody-conjugated protein-DNA complexes were precipitated by addition of protein A Sepharose beads (Santa Cruz), the immunoprecipitates were eluted, and the cross-links were reversed. The resulting DNAs were purified using QIAquick PCR purification kits (Qiagen) and analyzed by qPCR using iTaq universal SYBR green supermix (Biorad) and the Applied Biosystems prism real-time PCR system with the following primer pairs: BZLF1: *FWD*, 5′-GGCTGTCTATTTTTGACACCAGC-3′, and *REV*, 5-AAGGTGCAATGTTTAGTGAGTTACC -3′; and 4.8-kbps upstream of Zp transcription initiation site (negative control); *FWD*, 5′-AGAAGGGAGACACATCTG-3′, and *REV*, 5′-AACTTGGACGTTTTTGGG-3’. A standard curve was generated from the threshold cycle (*C*_*T*_) of the input DNA diluted to 5%, 1%, and 0.2% with distilled water containing 100 μg/ml sheared salmon sperm DNA (Ambion), with percent input bound calculated relative to this standard curve. Assays were performed in triplicate on two separate occasions.

### Construction of EBV mutant genomes

The 3-bp substitution mutations, HRE mt2 and HRE mt4, were introduced into the Zp HRE element in the EBV-containing BAC p2089 [36] by two-step, phage λ Red-mediated recombination essentially as previously described [80]. In brief, the I-SceI-Kan cassette present in pEPkan-S2 was PCR-amplified using the following primer pairs: HRE mt2: *FWD* 5’- AGGCATTGCTAATGTACCTCATAGACACACCTAAATTTAG***gct***GTCCCAAACCATGACATCACTAGGGATAACAGGGTAATCGATTT-3’ and *REV* 5’-CCAAGGCACCAGCCTCCTCTGTGATGTCATGGTTTGGGAC***agc***CTAAATTTAGGTGTGTCTATGCCAGTGTTACAACCAATTAACC-3’; HRE mt4: *FWD* 5’-AGGCATTGCTAATGTACCTCATAGACACACCTAAATTTAG***att***GTCCCAAACCATGACATCACTAGGGATAACAGGGTAATCGATTT-3’ and *REV* 5’-CCAAGGCACCAGCCTCCTCTGTGATGTCATGGTTTGGGAC***aat***CTAAATTTAGGTGTGTCTATGCCAGTGTTACAACCAATTAA-3’. The Zp sequence in these primers is underlined, with the base substitutions indicated in bold italicized small letters. These PCR products were electroporated into *E*. *coli* strain GS1783 into which BAC p2089 had been previously introduced, and inserted into p2089 by homologous recombination. Induction of the I-SceI activity encoded by GS1783 led to cleavage at the unique SceI site within the BAC. Intra-molecular recombination between the two copies of Zp resulted in precise removal of the inserted pEPkan-2 sequences, leaving behind one copy of Zp. Clones containing the desired HRE mutant BACs were initially identified by PCR screening and, subsequently, by DNA sequence analysis of the Zp and Zta-coding regions of the BAC. The mutant variants of p2089 were then thoroughly checked for absence of large deletions, insertions and rearrangements by analysis of multiple restriction enzyme fragment patterns as previously described [43,81] and for extraneous base-pair substitution mutations by high throughput sequence analysis as described below after recovery of the DNAs from mutant-infected 293T cell lines.

### Isolation of WT- and HRE mutant-infected 293T cell lines

293T cells were transfected with twice CsCl_2_-purified BAC DNA and selected for hygromycin-resistance as previously described [43]. By 3-to-4 weeks post-transfection, all of the colonies of cells were GFP-positive. These clones were picked, grown up, and stored in liquid nitrogen. Their ability to produce infectious virus was determined as previously described [35] following transfection with plasmids that express the EBV Zta and gp110 proteins. The titers of the mutant virus stocks ranged from 10^4^ to 10^5^ green Raji units (GRU)/ml.

### Sequence analysis of EBV HRE mutants

We recovered the BAC DNAs from the HRE mutant-infected 293T cell lines by Hirt extraction as previously described [43] and introduced them into *E*. *coli* strain GS500 by electroporation. Two independent colonies obtained from each of the two mutant BACs were grown, and the BAC DNAs were isolated by alkaline lysis as previously described [43]. After purification through two cycles of centrifugation in CsCl_2_, the highly purified BAC DNAs were sequenced using an Ion Torrent PGM (Life Technologies). We aligned the sequencing reads to the B95.8 reference strain of EBV (V01555) with Bowtie2 [82] using default alignment parameters and removing non-aligned reads. The resulting alignments were sorted using Samtools [83]. The Genome Analysis Toolkit (GATK) Unified Genotyper (https://www.broadinstitute.org/gatk/guide/article?id=6201) [84–86] was used to detect genetic variations compared to the EBV reference. Since regions of repetitive DNA produce incorrect alignments [87] which can manifest in downstream analyses as apparent mutations, we further investigated called mutations which occurred in the repetitive regions of the EBV genome (TRs, FRs, IR2, and IR3). A program termed EasyVariant was written and used to parse each alignment and its CIGAR string [83] that allowed both position-specific coverage depth to be calculated and percentage of each of the four nucleotides to be called at each position. Any position in which 50% or more reads indicated a mutation was treated as valid unless it occurred in a repeat region where it was likely due to an incorrect alignment. We achieved sequence coverage depth of 15 or more reads over 93% and 97% of the unique regions of the genome for HRE mt2 and mt4, respectively. The expected mutations in the HRE (mt2 and mt4) were called as such in 100% of sequence reads, and the consensus base calls within the unique regions of the genome matched the reference genome. We also performed conventional Sanger sequencing at four locations where some reads (but still less than 50%) indicated a possible frameshift mutation; in each of these cases, no mutation was found.

### Analysis of RNA expression data sets

SNU-719 transcript data, taken from Strong *et al*.[30], were analyzed using the RSEM algorithm (strand-specific option) for quantification of human gene expression [88] to calculate the relative levels of HIF-1α, HIF-2α, and HIF-3α RNA present in these cells. We likewise analyzed for relative expression of the HIF-αs the raw RNA-sequence reads obtained from four primary gastric carcinoma samples previously determined to contain high levels of EBV RNA [30]. These latter reads, generated through the NIH’s The Cancer Genome Atlas (TCGA) project, were obtained from the NCBI Sequence Read Archive (SRA035410, now available through the NCI Genomic Data Commons). The relative levels of the HIF-α RNAs present in primary, endemic, EBV^+^ Burkitt lymphomas were calculated from the data provided in Table S10 of Abate *et al*. [31]. The RNA expression levels of the genes shown in Fig 9A that had been generated from eight cell types ranging from naïve B cells to plasma cells (38 samples total) were retrieved from the previously reported microarray datasets [89–92]. These data were normalized using the GCRMA algorithm and visualized using GenomicScape (http://www.genomicscape.com/microarray/browsedata.php?acc=GS-DT-2) [93].

### Organotypic raft cultures

NOK (clone #3) cells were grown in organotypic culture as previously described [45] with the cells grown at the air-liquid interface for 11 days. Whole-cell extracts were prepared from these rafts by homogenization with a pestle in RIPA buffer [150 mM NaCl, 1% Triton X-100, 0.5% sodium deoxycholate, 0.1% SDS, 50 mM Tris (pH8.0)]. The resulting lysates were incubated on ice for 1/2 hour, sonicated, and centrifuged to remove debris. The supernatants were stored frozen until analyzed by immunoblotting.

### Analysis of EBV^+^ tumors generated in mice

EBV^+^ B-cell lymphomas were generated in immunodeficient NSG (NOD/LtSz-*scid/IL2Rγ*^*null*^) mice (Jackson Labs) as previously described [46]. In brief, CD34-depleted human cord blood mononuclear cells (#CB117; AllCells) were infected *in vitro* with the M81 strain of EBV (2,000 GRU) by incubation at 37°C for 1.5 h after which the infected cells were injected i.p. into 3- to 5-week-old NSG mice. Thirty-three days later, the mice were injected i.p. with 60 mg/kg of Hypoxyprobe (Hypoxyprobe) and sacrificed 1.5 h later by cervical dislocation under isoflurane anesthesia. Portions of the harvested tumors, along with some internal organs as controls, were submerged in Optimal Cutting Temperature compound and flash-frozen in ethanol-dry ice. Other portions of tumors, along with internal organs, were formalin-fixed and paraffin-embedded for sectioning and mounted onto slides for IHC.

EBV^+^ gastric cancer xenografts were generated by subcutaneous inoculation of 1x10^7^ SNU-719 cells in Matrigel into the flanks of NSG mice. Thirty-three days later, the mice were injected i.p. with 60 mg/kg of Hypoxyprobe and sacrificed 1.5 h later. Portions of the tumors were prepared as described above.

### Statistical analyses

*P*-values for the reporter assay data were determined by the Student’s t-Test method. The *p*-value for testing whether the distributions in Fig 12 were statistically different was determined by the Wilcoxon Rank-Sum Test using Mstat Statistical Software.

## Supporting information

S1 Fig IFS and IHC for Zta to determine efficiency of EBV reactivation by DFO in AGS-Akata and Sal cells. (A) AGS-Akata cells grown on cover slips were incubated for 24 h in the absence (-) or presence (+) of 200 μM DFO prior to fixing and processing for co-detection of Zta protein by IFS (green) and nuclei by staining with DAPI (blue). (B) AGS-Akata cells untreated (-) or treated (+) for 24 h with 200 μM DFO prior to harvesting, fixing, embedding in paraffin, sectioning, mounting on slides, and processing for co-detection of Zta by IHC (brown) and nuclei by counterstaining with hematoxylin (H; purple). (C) and (D), Sal cells were incubated with DFO and processed the same way as were the AGS-Akata cells in panels A and B, respectively. Brown arrows indicate locations of a few of the Zta^+^ cells.
(TIF)

S2 Fig HRE element is not a repressor-binding site and functions independently of the ZIIR element of Zp. (A) Basal activity levels observed with the Zp mutants in the reporter assays shown in Fig 5C. (B) Schematic showing sequence of the 6-bp HRE/ZIIR mutant analyzed in panel C. (C) Reporter assay showing failure of HIF-1α/β to activate transcription from a Zp mutant altered in the two 3’-most bases of the HRE along with the ZIIR element. Assays were performed as described in Fig 5C.
(TIF)

S3 Fig Adjacent serial sections of an M81-induced lymphoma stained for the indicated items. Protocol was the same as described in the legend to Fig 11. (A) Section co-stained for Zta (green) and Hypoxyprobe (red). (B) Section co-stained for Zta (green) and CD31 (red). (C) Section co-stained for EBNA2 (green) and CD31 (red). (D) Section stained with hematoxylin and eosin. Panels A-C were counterstained with DAPI (blue).
(TIF)

S4 Fig Most Zta-positive cells present in SNU-719 xenografts grown in NSG mice also reside distal to blood vessels. The flanks of NSG mice were inoculated with 1 x 10^7^ SNU-719 cells. Thirty-three days later, the mice were injected with Hypoxyprobe and sacrificed 1.5 h later. The tumors were flash frozen, sectioned, and stored at -80°C until processed by IFS as described in the legend to Fig 11. (A,B) Shown here are two representative sections co-stained for Zta (green) and CD31 (an endothelial marker indicative of blood vessels; red) and counterstained with DAPI (blue). Sections were photographed at the same magnification (40x).
(TIF)

S5 Fig Most Zta-positive cells present in B-cell lymphomas induced by EBV in humanized mice reside distal to blood vessels. NSG mice were inoculated i.p. with human cord blood that had been infected with the M81 strain of EBV. Thirty-three days later, the mice were sacrificed, and the tumors were processed by IHC for the indicated proteins. (A,B) Shown here are two representative sets of adjacent tumor sections stained for CD20, EBNA2, and Zta (brown) and with hematoxylin and eosin. These data are representative of data observed in over two dozen EBV^+^ tumors obtained in several experiments performed with cord blood from different donors. Purple and dark brown arrows point to locations of blood vessels and some of the Zta^+^ cells, respectively. Sections were photographed at the same magnification (40x).
(TIF)
