# Supplementary figures and images for: Hypoxia-inducible factor-1α plays roles in Epstein-Barr virus’s natural life cycle and tumorigenesis by inducing lytic infection through direct binding to the immediate-early BZLF1 gene promoter
Source: PLoS Pathog. 2017 Jun 15;13(6):e1006404. doi: 10.1371/journal.ppat.1006404 (PMC5487075; doi:10.1371/journal.ppat.1006404)

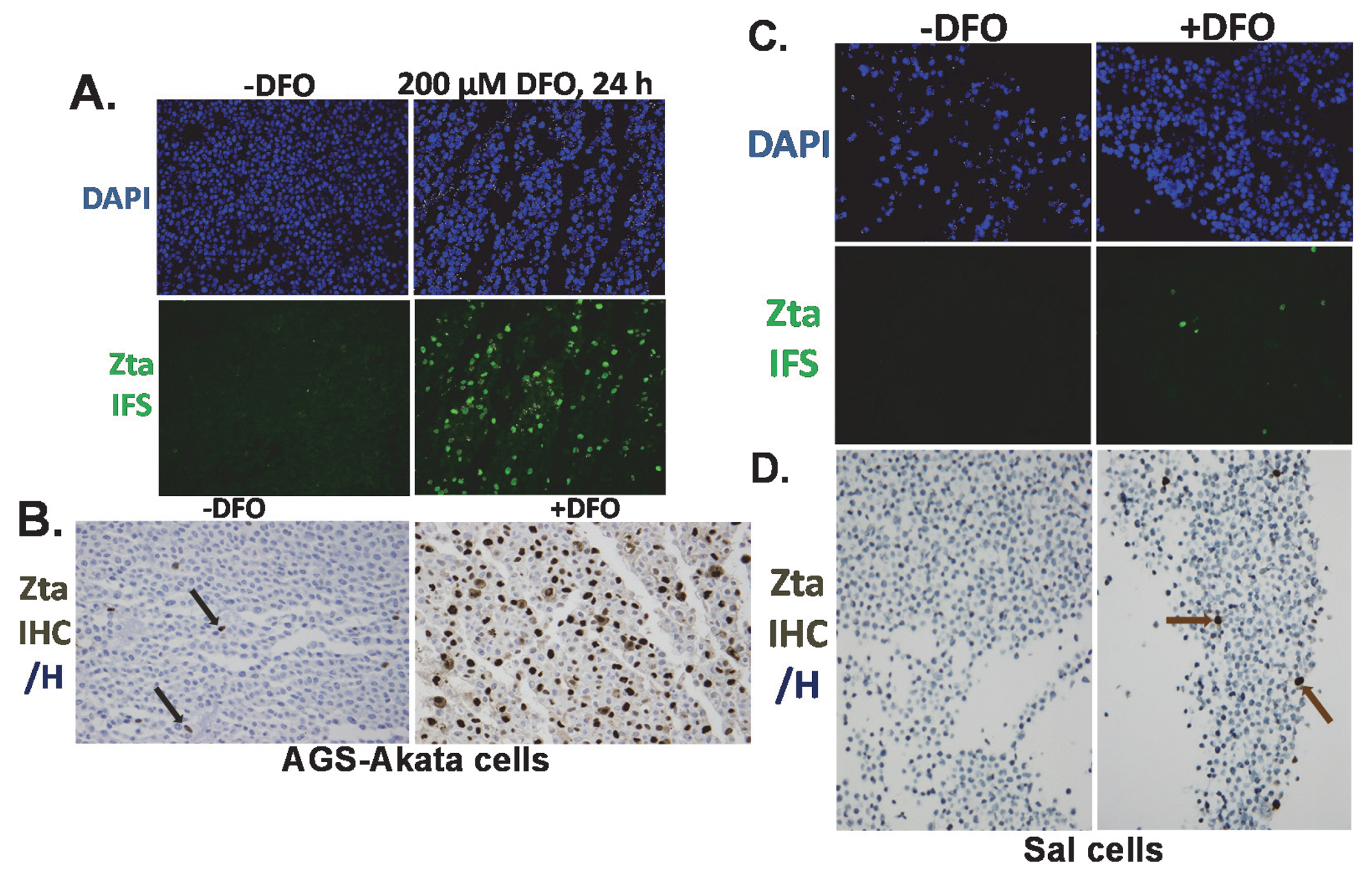

Supplement: S1 Fig — (A) AGS-Akata cells grown on cover slips were incubated for 24 h in the absence (-) or presence (+) of 200 μM DFO prior to fixing and processing for co-detection of Zta protein by IFS (green) and nuclei by staining with DAPI (blue). (B) AGS-Akata cells untreated (-) or treated (+) for 24 h with 200 μM DFO prior to harvesting, fixing, embedding in paraffin, sectioning, mounting on slides, and processing for co-detection of Zta by IHC (brown) and nuclei by counterstaining with hematoxylin (H; purple). (C) and (D), Sal cells were incubated with DFO and processed the same way as were the AGS-Akata cells in panels A and B, respectively. Brown arrows indicate locations of a few of the Zta+ cells. (TIF) [file ppat.1006404.s001.tif]

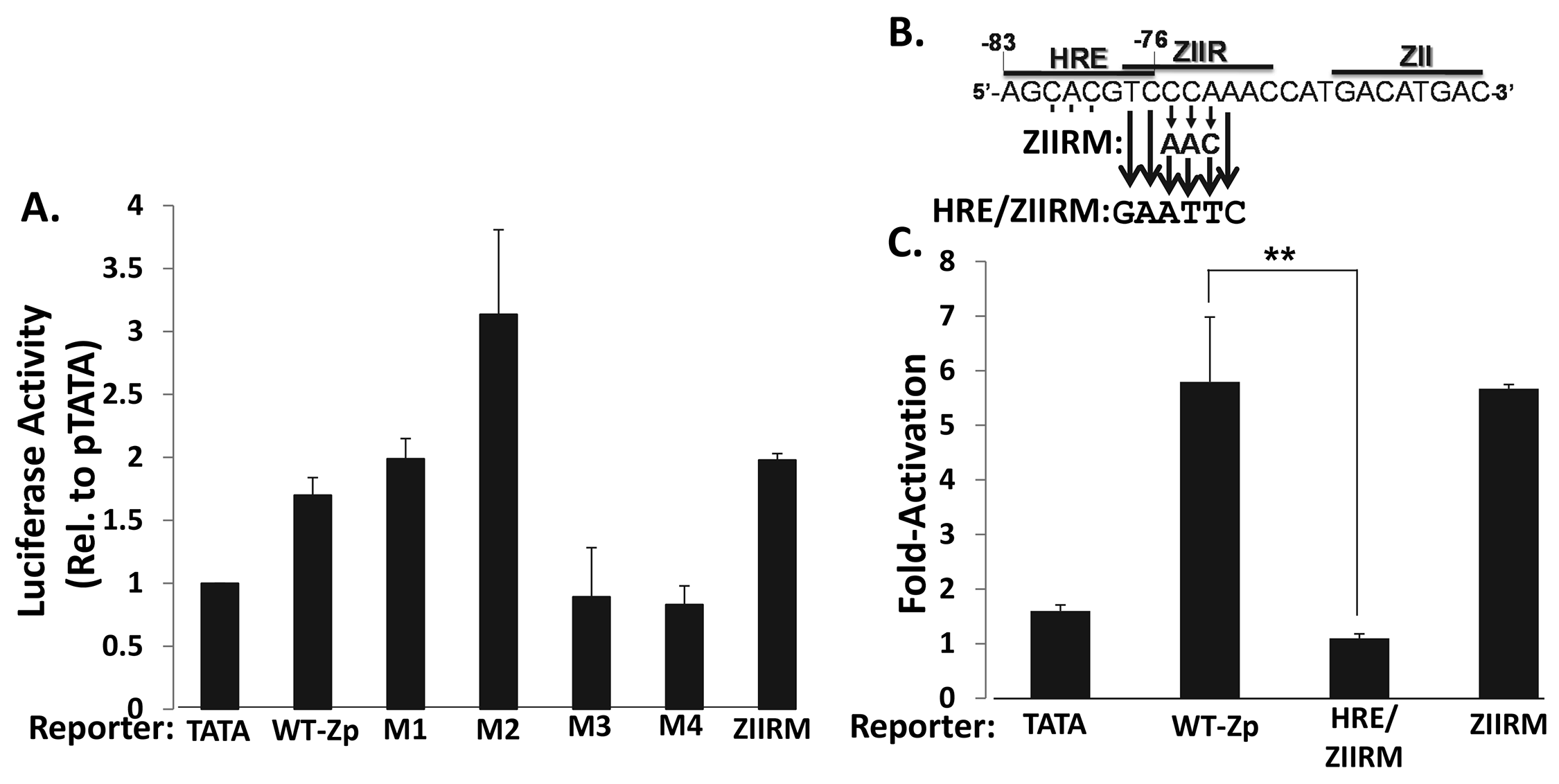

Supplement: S2 Fig — (A) Basal activity levels observed with the Zp mutants in the reporter assays shown in Fig 5C. (B) Schematic showing sequence of the 6-bp HRE/ZIIR mutant analyzed in panel C. (C) Reporter assay showing failure of HIF-1α/β to activate transcription from a Zp mutant altered in the two 3’-most bases of the HRE along with the ZIIR element. Assays were performed as described in Fig 5C. (TIF) [file ppat.1006404.s002.tif]

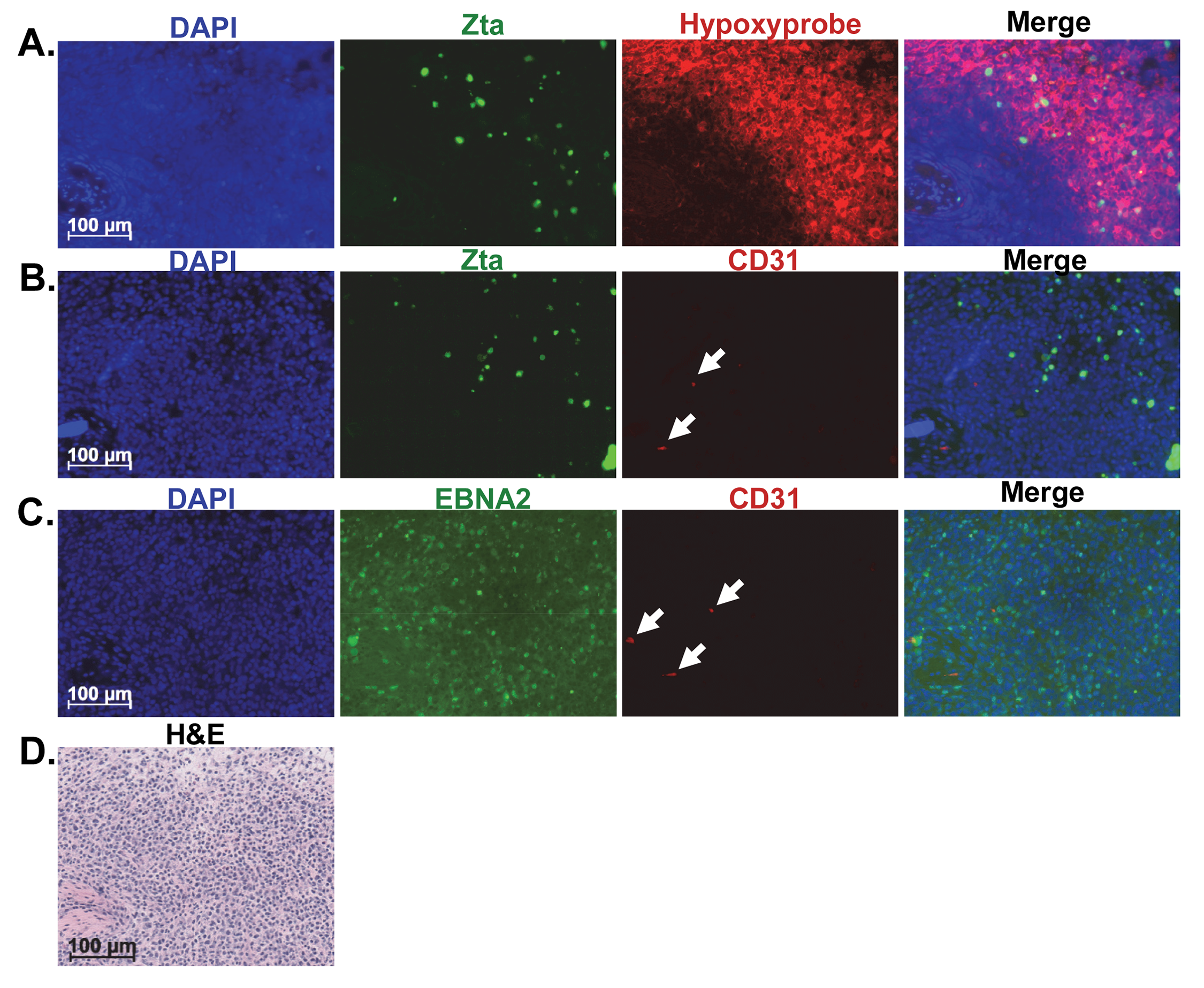

Supplement: S3 Fig — Protocol was the same as described in the legend to Fig 11. (A) Section co-stained for Zta (green) and Hypoxyprobe (red). (B) Section co-stained for Zta (green) and CD31 (red). (C) Section co-stained for EBNA2 (green) and CD31 (red). (D) Section stained with hematoxylin and eosin. Panels A-C were counterstained with DAPI (blue). (TIF) [file ppat.1006404.s003.tif]

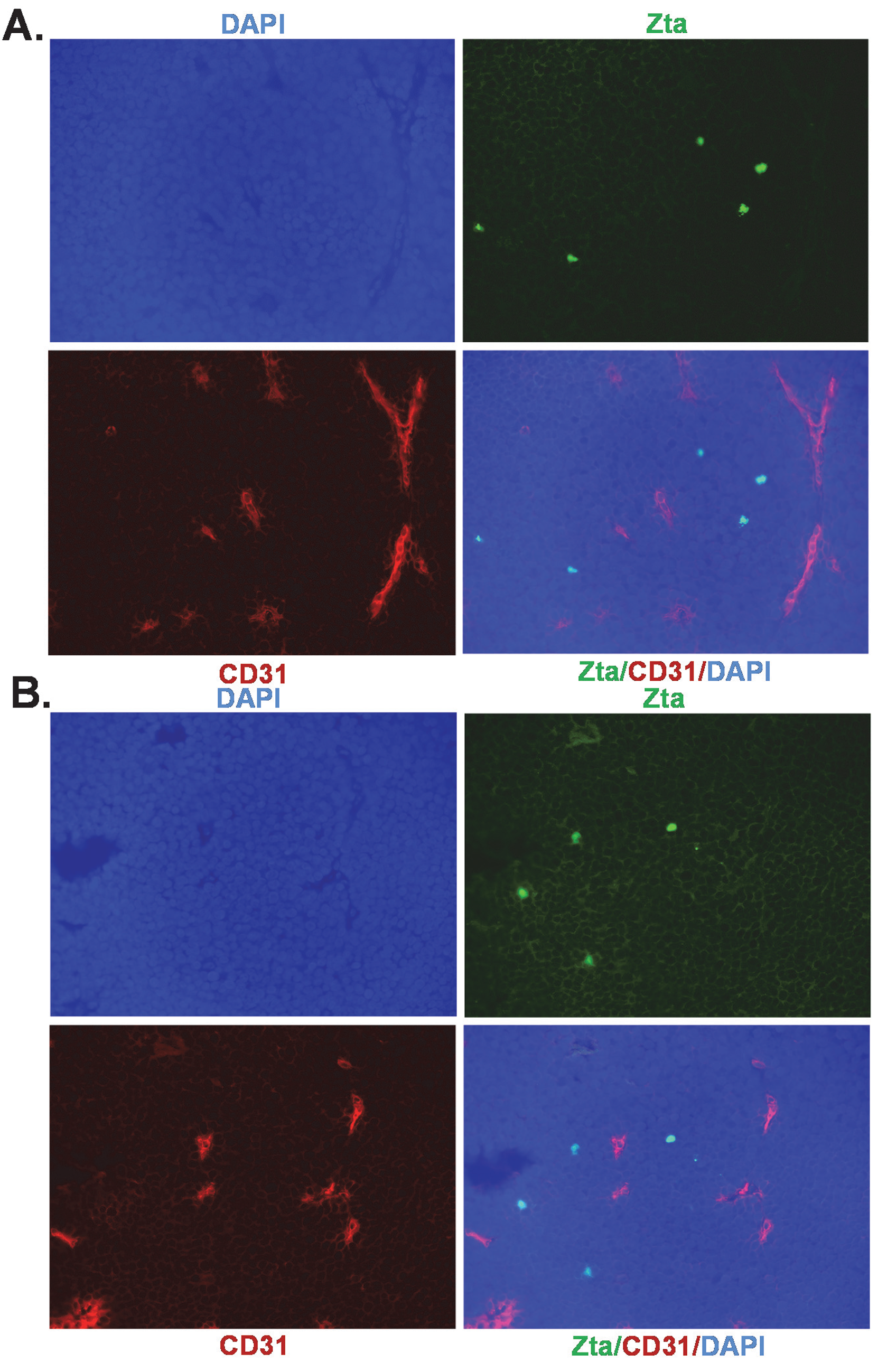

Supplement: S4 Fig — The flanks of NSG mice were inoculated with 1 x 10^7^ SNU-719 cells. Thirty-three days later, the mice were injected with Hypoxyprobe and sacrificed 1.5 h later. The tumors were flash frozen, sectioned, and stored at -80°C until processed by IFS as described in the legend to Fig 11. (A,B) Shown here are two representative sections co-stained for Zta (green) and CD31 (an endothelial marker indicative of blood vessels; red) and counterstained with DAPI (blue). Sections were photographed at the same magnification (40x). (TIF) [file ppat.1006404.s004.tif]

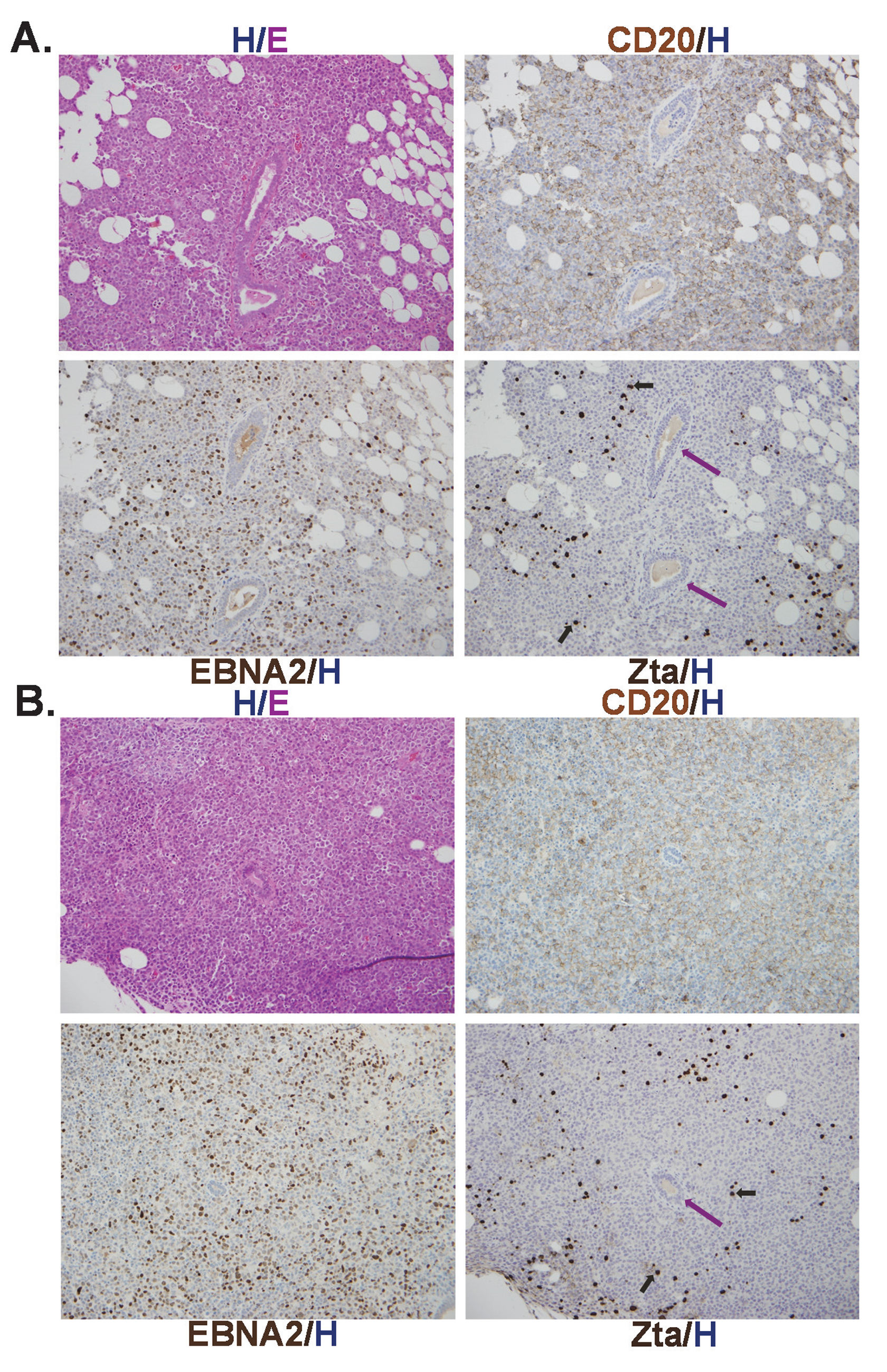

Supplement: S5 Fig — NSG mice were inoculated i.p. with human cord blood that had been infected with the M81 strain of EBV. Thirty-three days later, the mice were sacrificed, and the tumors were processed by IHC for the indicated proteins. (A,B) Shown here are two representative sets of adjacent tumor sections stained for CD20, EBNA2, and Zta (brown) and with hematoxylin and eosin. These data are representative of data observed in over two dozen EBV+ tumors obtained in several experiments performed with cord blood from different donors. Purple and dark brown arrows point to locations of blood vessels and some of the Zta+ cells, respectively. Sections were photographed at the same magnification (40x). (TIF) [file ppat.1006404.s005.tif]
